# Systematic profiling of AGO2-associated circRNAs and functional investigation reveal circN4BP2L2 as a survival-promoting regulator in T-ALL

**DOI:** 10.1038/s41375-026-03033-x

**Published:** 2026-07-27

**Authors:** Monika Drobna-Sledzinska, Alessia Buratin, Eleonora Roncaglia, Alberto Caregari, Ilias Glogovitis, Maria Kosmalska, Xing Zhao, Silvia Bresolin, Enrico Gaffo, Anke Van den Berg, Joost Kluiver, Malgorzata Dawidowska, Stefania Bortoluzzi

**Affiliations:** 1 Institute of Human Genetics, Polish Academy of Sciences, Poznan, Poland; 2 Department of Molecular Medicine, University of Padova, Padova, Italy; 3 Department of Biology, University of Padova, Padova, Italy; 4 Department of Oncology Surgery and Gastroenterology, University of Padova, Padova, Italy; 5 Department of Pathology and Medical Biology, University of Groningen, University Medical Centre Groningen, Groningen, the Netherlands; 6 Onco-Hematology, Stem Cell Transplant and Gene Therapy Laboratory, IRP-Istituto Di Ricerca Pediatrica, Padua, Italy

**Keywords:** Acute lymphocytic leukaemia, Cancer genomics, Apoptosis, Oncogenes

## Abstract

Circular RNAs constitute an emerging area of intensive research in cancer. In T-cell acute lymphoblastic leukemia (T-ALL), an aggressive hematologic malignancy characterized by clonal proliferation of T-cell precursors, the function of most aberrantly expressed circRNAs is not yet understood. To identify circRNAs acting as miRNA sponges, we performed AGO2 immunoprecipitation and RNA sequencing (AGO2-RIP-seq) in four T-ALL cell lines. Our analysis revealed circRNAs consistently enriched in the AGO2-bound fraction, highlighting a new resource for T-ALL research. Notably, circRNAs were more enriched (7.3%) compared to linear RNAs (3%). For functional investigation, we focused on the most abundant AGO2-bound circRNAs that were also found to be dysregulated in T-ALL patients. Knockdown of circN4BP2L2 revealed its ability to promote cell proliferation and confer resistance to apoptosis in T-ALL cell lines, in addition to modulating key T-ALL pathogenic pathways. Analysis of gene expression of T-ALL patients disclosed peculiar features of T-ALL with high circN4BP2L2 expression. A marked overlap was observed between differentially expressed genes upon knockdown of circN4BP2L2 in vitro and the profiles of circN4BP2L2 stratified T-ALL cases. This work provides new insights into the circRNA-mediated regulatory networks driving this malignancy and presents circN4BP2L2 as a key RNA shaping oncogenic phenotype in T-ALL.

## Introduction

T-cell acute lymphoblastic leukemia (T-ALL) is an aggressive hematologic malignancy characterized by the clonal proliferation of immature T-cell precursors. Although it accounts for a minority of pediatric and adult ALL cases, T-ALL is clinically significant due to rapid progression, frequent resistance to conventional therapies, and poor prognosis in refractory and relapsed cases [1, 2].

Genomic and transcriptomic profiling have uncovered a range of alterations and signaling pathways frequently involved in T-ALL pathogenesis, including aberrations in NOTCH1, NOTCH3, CDKN2A/B, and the PI3K-AKT pathway [3, 4]. Despite these insights, the complex molecular landscape of T-ALL and its marked inter- and intra-patient heterogeneity remains incompletely understood.

Seminal studies have clarified that recurrent genetic aberrancies and driver variants cooperate in causing the malignant transformation and differentiation block of thymocytes. A more in-depth understanding of T-ALL at the molecular level came from transcriptomic studies that disclosed disease mechanisms and revealed new subtypes [5–7]. The inclusion of whole genome sequencing revealed another feature of the complex T-ALL biology – approx. 60% of known oncogenic drivers are linked to alterations in the noncoding genome [7]. Research focused on alterations affecting non-coding RNAs opens new resources for the investigation of T-ALL biology, with potential clinical implications [8–10]. Circular RNAs (circRNAs) are increasingly recognized as versatile regulators of cellular functions, with direct involvement in oncogenic axes, by several mechanisms [11–16]. Their best-described function is their action as competing endogenous RNAs (ceRNAs), which de-repress other transcripts by sequestering shared microRNAs, thereby shaping oncogenic networks [15, 16]. CircRNAs’ stability makes this class of molecules a promising source of biomarkers [17–19].

In T-ALL, the role of circRNAs has only recently started to be explored. The oncogenic potential of circPVT1 [20] and circZNF609 [21] was indicated by in vitro studies. Functional in vitro investigation and transcriptomics analysis of patient samples demonstrated circFBXW7 as a tumor suppressor in this malignancy [22].

In pediatric T-ALL, we revealed dysregulation of circRNAs and identified subtype-specific circRNA signatures across five molecular subtypes [21]. Moreover, we showed that downregulation of the QKI splice factor in a subset of T-ALL cases is associated with a distinct circRNA expression profile [23], indicating the potential of circRNA studies to explain T-ALL heterogeneity.

Data published so far show that several circRNAs are aberrantly expressed in T-ALL, but only a few have been functionally characterized. Driven by these knowledge gaps, we aimed to investigate the function of circRNAs overexpressed in T-ALL. Using AGO2-IP, we identified circRNAs potentially engaging in miRNA-related regulatory networks. We focused on AGO2-IP-enriched circRNAs with dysregulated expression in T-ALL patients. We demonstrated that circN4BP2L2, generated by backsplicing of exons 3–6 of the *N4BP2L2* gene, is enriched in the AGO2-containing RNA-induced silencing complex (RISC), and has oncogenic properties in T-ALL in vitro. Exploration of the transcriptomic profile of a large cohort of pediatric T-ALL patients stratified according to circN4BP2L2 expression disclosed distinct molecular features of circN4BP2L2-high T-ALL cases. Knockdown of this circRNA in T-ALL cell lines reversed the molecular profile characteristic of circN4BP2L2-high cases.

## Materials and methods

### AGO2 RNA immunoprecipitation (AGO2-IP)

AGO2-IP was performed in four T-ALL cell lines DND-41, ALL-SIL, JURKAT and LOUCY (Supplementary Information). For each immunoprecipitation, 3 × 10^7^ cells were used. AGO2-IP was performed as described previously [24]. Briefly, cell lysates were incubated with EZview Red Protein G Affinity Gel (Merck, Darmstadt, Germany) coated with anti-AGO2 antibody (Clone 2E12-1C9, Abnova) at 4 °C overnight. Anti-IgG antibody was used as a negative control (Merck). 10% of input, AGO2-IP, and flow-through fractions were collected to test the effectiveness of the procedure via immunoblotting and RT-qPCR for selected miRNAs. The remaining AGO2-IP fraction was used for RNA isolation, quality control, and sequencing (Supplementary Information).

### RNAse R treatment, RT-qPCR and subcellular fractionation

To test the effectiveness of AGO2-IP, enrichment of three miRNAs abundant in T-ALL cell lines (hsa-miR-16-5p, hsa-let-7a-5p, and hsa-miR-25-3p) [25] was evaluated (Supplementary Information). For circRNA quantification, total RNA was reverse transcribed using iScript (Bio-Rad, Hercules, CA, USA) and amplified using HOT FIREPol EvaGreen qPCR Mix Plus (Solis Biodyne, Tartu, Estonia). The primers used for RT-qPCR and RT-PCR are listed in Supplementary Table S1 and Supplementary Table S2. For RNAse R treatment, Ribonuclease R (Lucigen, Middlesex, UK) was used according to the manufacturer’s protocol. The details of subcellular fractionation are described in Supplementary Information.

### Detection and quantification of circRNAs and linear transcripts

The RNA-seq data have been analyzed with the CirComPara2 bioinformatics pipeline [26, 27] to quantify both gene and circRNA expression. Details on circRNA filtering and annotation, calculation of the circular-to-linear proportion (CLP) [21], expression data normalization and correction and saturation analysis are reported in Supplementary Information.

### Enrichment score calculation

The generalized fold change (gfold) [28] of the observed backsplice junction (BSJ) reads number was used as a measure of circRNA abundance difference between AGO2-IP and total (TOT) RNA fractions. A detailed mathematical description of the Bayesian statistical framework underlying gfold can be found in Supplementary Information. Briefly, gfold functions similarly to a conventional fold change but accounts for the statistical significance of the observation, offering an effective means to rank enriched transcripts using a p-value threshold.

### Transduction of T-ALL cell lines for circRNA silencing

shRNA-encoding sequences (reverse complement to the BSJ region) were cloned into a miRZip pGreenPuro vector (System Biosciences). The sequences of shRNA oligonucleotides are listed in Supplementary Table S3. As a control, miRZip pGreenPuro non-targeting (NT) vector was used (System Biosciences, Palo Alto, CA, UK). For lentiviral particle assembly, pRSV.REV, pMSCV-VSV-G, and pMDLg/PRRE vectors (Addgene) were used. The detailed procedure and subsequent functional assays are described in the Supplementary Information.

### Gene expression analysis upon circN4BP2L2 silencing and miRNA-binding predictions

Gene expression was quantified from mRNA-seq data generated upon circN4BP2L2 silencing in cultured cells using CircComPara2 [26]. Differential expression and gene set enrichment analyses were performed on 12637 expressed genes. Analysis details are provided in Supplementary Information.

To predict circN4BP2L2-miRNA interactions, we considered 588 human miRNAs expressed in T-ALL according to small RNA-seq data [10]. Predictions of miRNA binding sites in the circN4BP2L2 sequence were obtained using four tools (MiRanda, TargetScan, IntaRNA, and PITA), and stringent criteria to keep only high confidence predictions [29] (Supplementary Information).

### Statistical analysis

All statistical analyses were performed in R (v4.3.0). Comparisons between groups were performed using the Wilcoxon rank-sum test for continuous variables and Fisher’s exact test for categorical variables. Multiple testing correction was applied using the Benjamini-Hochberg (BH) procedure; adjusted *p* values < 0.05 were considered statistically significant.

### T-ALL patients’ data analysis

Gene and circRNA expression was quantified using the CircComPara2 in 242 samples of a pediatric T-ALL cohort profiled by total RNA-seq (dbGaP Study Accession: phs000464.v13.p6) and annotated with molecular subtypes. Filtering out low-abundant genes (mean transcript per million TPM < 1), 14,607 genes were kept.

## Results

### AGO2-RIP-seq identifies circRNAs potentially interacting with miRNAs in T-ALL in vitro

To explore the role of circRNAs in T-ALL as ceRNAs and to identify circRNAs putatively interacting with miRNAs, we performed AGO2-RIP-seq in four T-ALL cell lines, representing different genetic subtypes, i.e., JURKAT (TAL1), ALL-SIL (TLX1), DND-41 (TLX3), and LOUCY (ETP-ALL). AGO2 is an essential component of the RISC, to which RNAs are driven by interacting miRNAs.

**Fig. 1:**
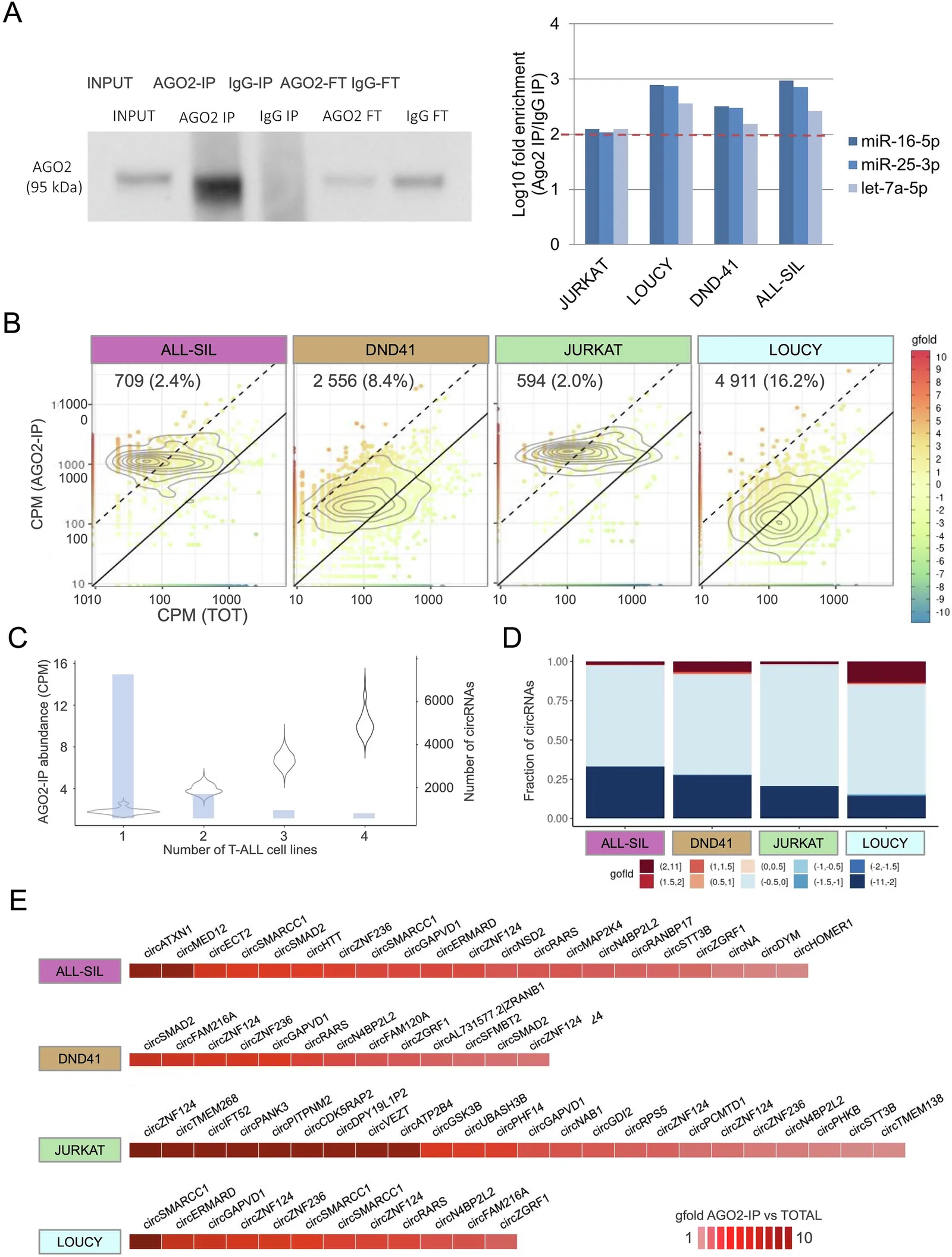
Circular RNAs enriched in the AGO2-IP fraction in T-ALL cell lines. **A** Verification of the efficiency of the AGO2 RNA immunoprecipitation procedure using Western Blot (left panel, exemplary result obtained for JURKAT cells) and RT-qPCR for 3 selected miRNAs in 4 T-ALL cell lines (right panel; IgG - isotype control; IP - immunoprecipitation fraction; FT - flow-through fraction; Fold enrichment of miRNAs in AGO2-IP fraction in reference to IgG-IP fraction was calculated as 2-ΔCq (Cq–quantification cycle), where ΔCq = Cq(AGO2-IP) – Cq(IgG-IP). **B** Density contour plot of circRNA abundance in the AGO2-IP and in the TOTAL fractions per cell line (the dot color indicates the magnitude of enrichment given by gfold value, the bisector and dashed line of enrichment threshold with gfold at least 1 are shown, for each cell line). **C** Violin plot of average abundance in the AGO2-IP fraction of circRNAs binned by number of cell lines in which they were detected; the overlapping bar plot shows the number of circRNAs per class. **D** Fraction of circular transcripts per gfold comparing AGO2-IP fraction vs TOTAL fraction in the four T-ALL cell lines. **E** The most abundant and enriched circRNAs in the AGO2-IP fraction for each cell line (only circRNAs with abundance at least 3000 CPM are shown; the enrichment is indicated by the gfold square color).

The efficiency of RISC pulldown via AGO2-IP was confirmed using two approaches (Fig. 1A). Immunoblotting with anti-AGO2 antibody demonstrated clear enrichment of AGO2 in IP samples. In parallel, RT-qPCR analysis of three representative miRNAs revealed a strong enrichment in AGO2-IP fractions compared to the negative control. On average, a 411-fold enrichment of the tested miRNAs was achieved, ranging from 120-fold in JURKAT to 640-fold in ALL-SIL.

CirComPara2 analysis of RNA-seq data of AGO2-IP and TOT RNA samples in four cell lines detected 34773 reliable circRNAs, derived from 7839 host genes. In the same sample set, a total of 124898 linear transcripts were detected. As expected, only a subset of RNAs was found in AGO2-IP samples (Fig. 1B, Supplementary Fig. S1A and Supplementary Fig. S2). Unsupervised multidimensional scaling analysis (MDSMSA) of circRNA abundance indicated a distinct circRNA profile in the AGO2-IP fraction, with a clear separation from the TOT samples (Supplementary Fig. S1B). RNA-seq saturation analysis confirmed that the AGO2-IP and TOT fraction sequencing depth was adequate for circRNA expression analysis (Supplementary Fig. S3). CircRNA abundance was higher in AGO2-IP than in TOT fraction, in all cell lines (Supplementary Fig. S4). Density contour plots of circRNA abundance in AGO2-IP versus TOT reveal a shift of the distribution toward circRNA enrichment in the AGO2-bound fraction (Fig. 1B). In the AGO2-IP samples, circRNA abundance ranged in three orders of magnitude (from 0 to ~35000 CPM). Fig. 1C shows the number and the expression distribution of circRNAs detected in one to all cell lines. The circRNAs most shared between cell lines had higher abundances.

Quantitative comparison of RNAs’ abundance in the AGO2-IP vs the TOT samples per cell line indicated the circRNAs that were significantly enriched in the AGO2-IP fraction, and thus potentially interacting with miRNAs in vitro. We adopted a Bayesian approach to assign a *p* value to the observed abundance change (gfold) of circRNAs and linear transcripts between fractions, taking into account the uncertainty of expression measures in the two conditions being compared. We considered enriched in the AGO2-IP samples only RNAs with an estimated gfold of at least 1, corresponding to a P-adj ≤0.05 (Fig. 1D, E). We found 8770 circRNAs enriched in the AGO2-IP fraction of at least one cell line. On average, 7.3% of the circRNAs were enriched in the AGO2-IP fraction, according to the stringent criteria used, with per cell line values ranging from 2.4% (JURKAT) to 16.2% (LOUCY) (Fig. 1D). Interestingly, a larger fraction of circular than linear RNAs appeared significantly enriched in the AGO2-IP samples. Only 3% of linear RNAs on average were enriched (Supplementary Fig. S5**)** and showed a gfold distribution more dense around zero than that of circRNAs (Kolmogorov-Smirnov test, p-value < 0.001, Supplementary Fig. S6).

**Fig. 2:**
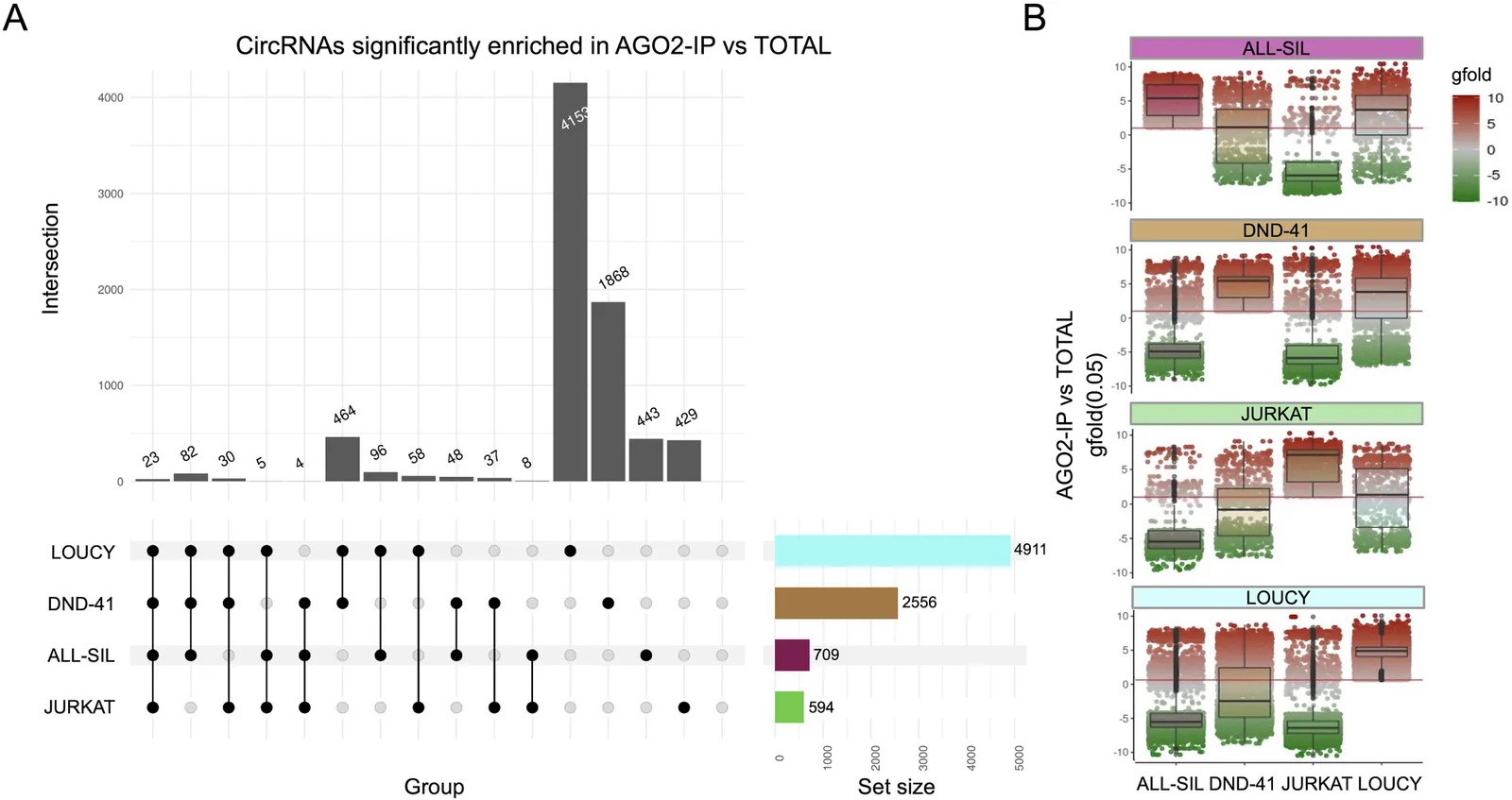
Definition of circular RNAs enriched in the AGO2-IP fraction in multiple T-ALL cell lines. **A** UpSet plot showing circRNAs significantly enriched in the AGO2-IP fraction in different cell lines. The horizontal bars on the right indicate the total number of circRNAs significantly enriched per cell line, the vertical bars represent the number of circRNAs shared across intersections of two or more cell lines (indicated by connected dots below the bars). **B** gfold distribution of circRNAs significantly enriched in the AGO2-IP fraction, across cell lines. For each cell line, a box plot with overlaid dot plots shows the gfold values of circRNAs; In the different plots, laid in vertical, circRNAs enriched in the cell line indicated in the title are shown; Dots are colored by gfold direction and magnitude, from green (depleted) to red (enriched).

The circRNAs enriched in the AGO2-IP fraction with high abundance (≥3000 CPM) are shown in Fig. 1E. We found 855 circRNAs significantly enriched in the AGO2-IP fraction concordantly in at least two cell lines, indicating a robust association with AGO2-containing complexes and supporting their involvement in miRNA-mediated regulation. On top, 144 circRNAs were enriched in three and 23 in all four analyzed cell lines (Fig. 2A and Supplementary Table S4). Figure 2B shows the overall degree of concordance between cell lines, indicating, for each cell line, the gfold of circRNAs enriched in that and also in the other cell lines.

### AGO2-IP-enriched circRNAs with aberrant expression in T-ALL: selection and validation

**Fig. 3:**
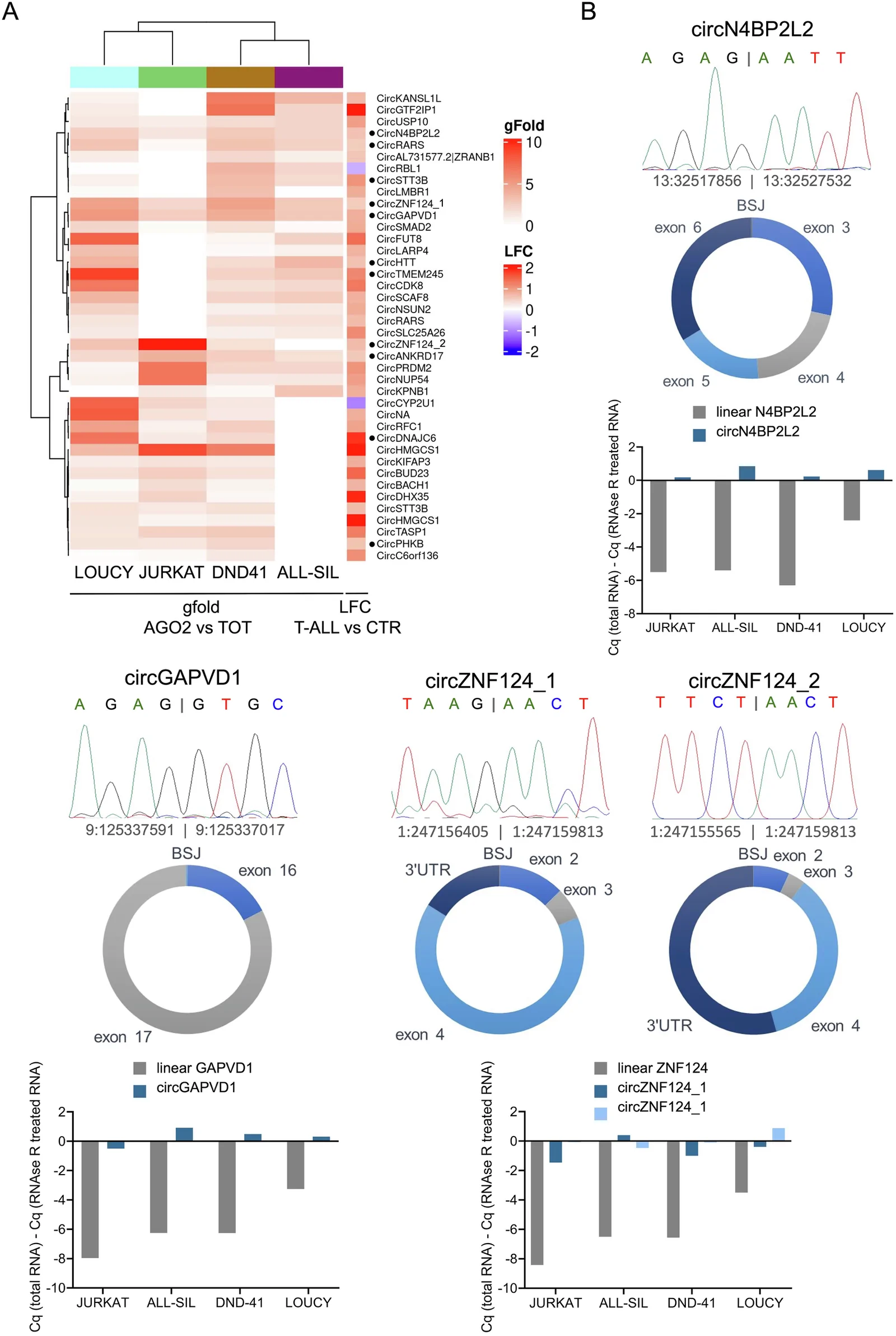
Circular RNAs enriched in the AGO2-IP fraction in T-ALL cell lines and upregulated in T-ALL. **A** Heatmap of selected circRNAs aberrantly expressed in T-ALL patients and enriched across multiple cell lines. A curated panel of circRNAs showing aberrant expression in T-ALL and recurrent enrichment in AGO2-IP across cell lines is shown; The heatmap displays gfold values for each circRNA in individual cell line AGO2-IP vs control comparisons; Rows represent circRNAs, while columns and different colors above represent cell lines, with a total of 31 circRNAs enriched in three cell lines and nine circRNAs enriched in all four; On the right the log2 fold change (LFC) in T-ALL patient samples compared with control samples is reported; CircRNAs selected for experimental validation are indicated by black dots. **B** Validation of circRNA sequence and circular nature; Sanger sequencing chromatograms showing the backsplice junction of each circRNA, with a schematic representation of their circular structures and included exons; lower panel, RT-qPCR analysis of circular and linear transcripts in four T-ALL cell lines following RNase R treatment, confirming resistance of circular RNA to exonuclease digestion.

We focused on AGO2-IP-enriched circRNAs aberrantly expressed in T-ALL patients. Among circRNAs previously reported as dysregulated in the T-ALL pediatric cohort compared with normal thymocytes [21], 299 were enriched in the AGO2-IP fraction of at least one T-ALL cell line according to our data, the majority (285) being overexpressed in T-ALL. On top, 31 circRNAs aberrantly expressed in T-ALL patients were enriched in the AGO2-IP fraction in at least three cell lines (Fig. 3A). Among these, we selected for validation of their circular nature and expression eleven circRNAs with relatively high abundance in T-ALL (mean read count in AGO2-IP fraction >1000). We designed sets of divergent primers to amplify regions spanning the BSJ using cDNA from cell lines. Sanger sequencing of the resulting PCR products allowed reconstruction of the full circRNA sequences and validation of the BSJ identified by RNA-seq. Next, we tested the resistance of circRNAs to RNase R, the gold standard method to assess the circularity of transcripts. By comparing the RT-qPCR expression estimate in RNAse R-treated and non-treated RNA from T-ALL cell lines, we validated expression and circularity in eight circRNAs: circGAPVD1, circZNF124_1(1:247156405-247159813:-), circZNF124_2 (1:247155565-247159813:-), circN4BP2L2 (Fig. 3B), circRARS, circHTT, circTMEM245, circSTT3B (Supplementary Fig. S7). For circPHKB, circANKRD17, and circDNAJC6, the validation was unsuccessful due to a lack of detectable amplicon.

### Functional experiments disclosed that circN4BP2L2 sustains T-ALL growth and survival in vitro

**Fig. 4:**
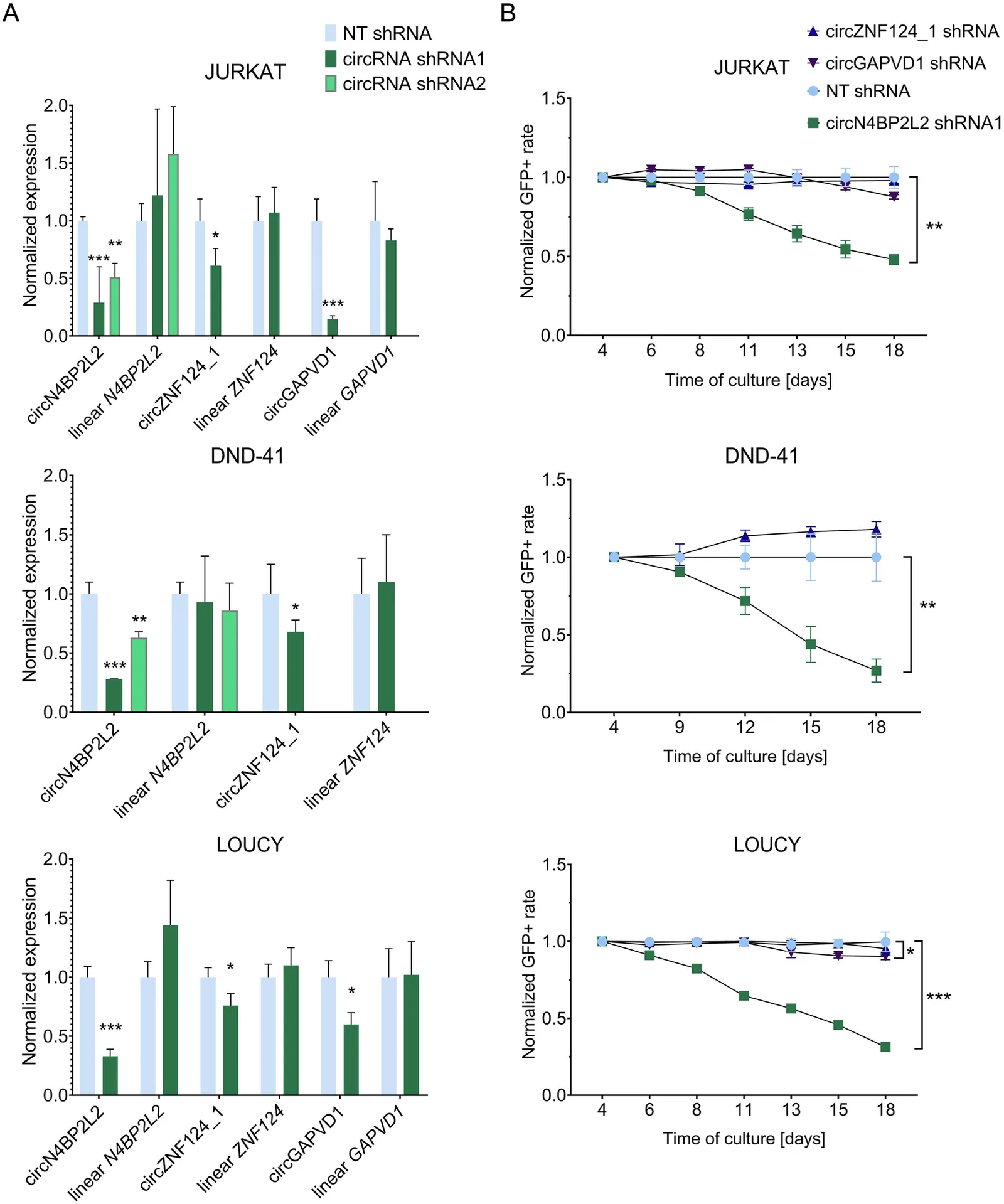
Evaluation of the effects of circRNA silencing on survival advantage in T-ALL cell lines. **A** Evaluation of the expression of circular and linear transcripts upon the use of shRNA targeting BSJ of selected circRNAs compared to control (non-targeting shRNA) via RT-qPCR in JURKAT, DND-41, and LOUCY cell lines, analyzed with an unpaired two-tailed *t* test. **B** GFP growth competition assay upon repression of selected circRNA in JURKAT, DND-41, and LOUCY T-ALL cell lines. The GFP percentage for each time point was calculated in reference to the GFP percentage at the first day of measurement (Day 4 after transduction), and analyzed with two-way ANOVA (* *p* < 0.05; ** *p* < 0.01; *** *p* < 0.001).

We brought to functional investigation the three validated circRNAs that were AGO2-IP-enriched in all four cell lines. As previously mentioned, all of them were upregulated in T-ALL patient samples. We obtained specific inhibition of circZNF124_1, circGAPVD1, and circN4BP2L2 in vitro, in JURKAT, DND-41, and LOUCY cells using shRNA targeting the unique BSJ region of each circRNA. The average effectiveness of repression was ~40% for circZNF124, 45–70% for circN4BP2L2, and 45–85% for circGAPVD1 (Fig. 4A). The silencing of circRNAs did not affect the expression of corresponding linear transcripts.

We next investigated the effect of the silencing of each circRNA on cell growth by performing a GFP competition assay (Fig. 4B). No significant advantage or disadvantage was observed upon circZNF124 compared to control cells. CircGAPVD1 silencing resulted in significant but very modest growth disadvantage only in LOUCY cells. Instead, a significant and sizable growth disadvantage was observed in all the three tested cell lines upon repression of circN4BP2L2.

**Fig. 5:**
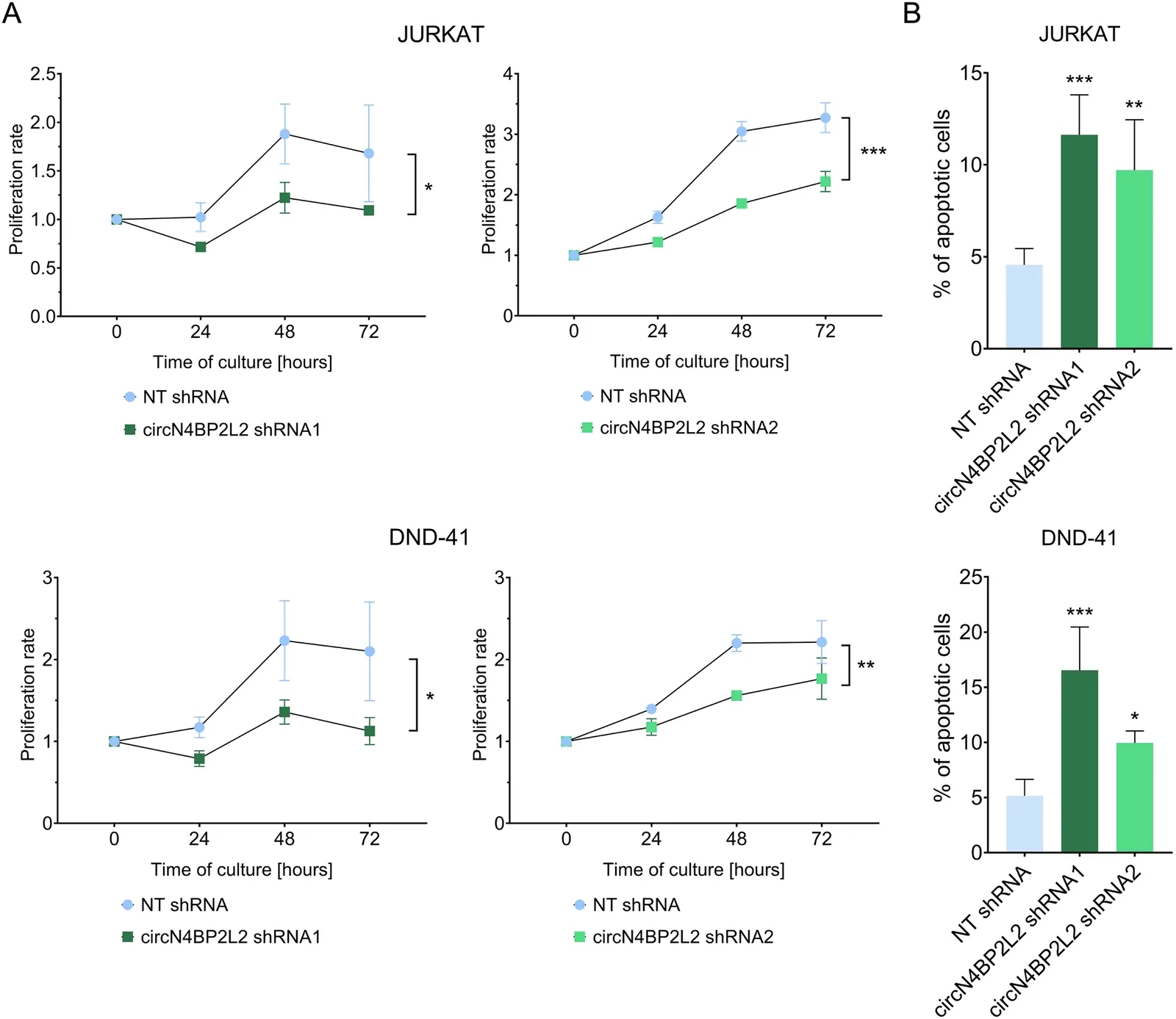
Evaluation of proliferation and apoptosis in T-ALL cell lines upon silencing of circN4BP2L2. **A** Cell proliferation measured by colorimetric viability assay in JURKAT and DND-41 cell lines. Proliferation rate was calculated as fold change of OD450 for each examined time point in reference to the starting point (0 h of culture, 4 days after transduction). **B** Percentage of apoptotic cells evaluated 5 days after transduction of JURKAT and DND-41 cell lines with non-targeting shRNA (control), circN4BP2L2 shRNA1 or shRNA2 by Annexin-V APC staining and flow cytometry (* - *p* < 0.05; ** - *p* < 0.01; *** - *p* < 0.001).

We showed a predominant cytoplasmic location of this circRNA in T-ALL cell lines (Supplementary Fig. S8). Next, we examined the influence of this circRNA on the proliferation of JURKAT and DND-41 using two different shRNAs. We observed decreased cell proliferation capacity upon circN4BP2L2 knockdown, evidenced by a lower fold increase in cell number compared to control over 48 h of culture, despite both conditions reaching a stationary phase between 48 and 72 h. Moreover, viable cell number decreased upon circN4BP2L2 knockdown with shRNA1 after 24 hours of culture compared to the starting point, indicating cell death (Fig. 5A). Apoptosis assay, performed at the same timepoint, showed a significant increase in the percentage of apoptotic cells upon the knockdown with both shRNAs. This points to an anti-apoptotic role of circN4BP2L2 in T-ALL in vitro (Fig. 5B, Supplementary Fig. S9).

### Transcriptomic analysis reveals the impact of circN4BP2L2 silencing on gene expression and regulatory networks

**Fig. 6:**
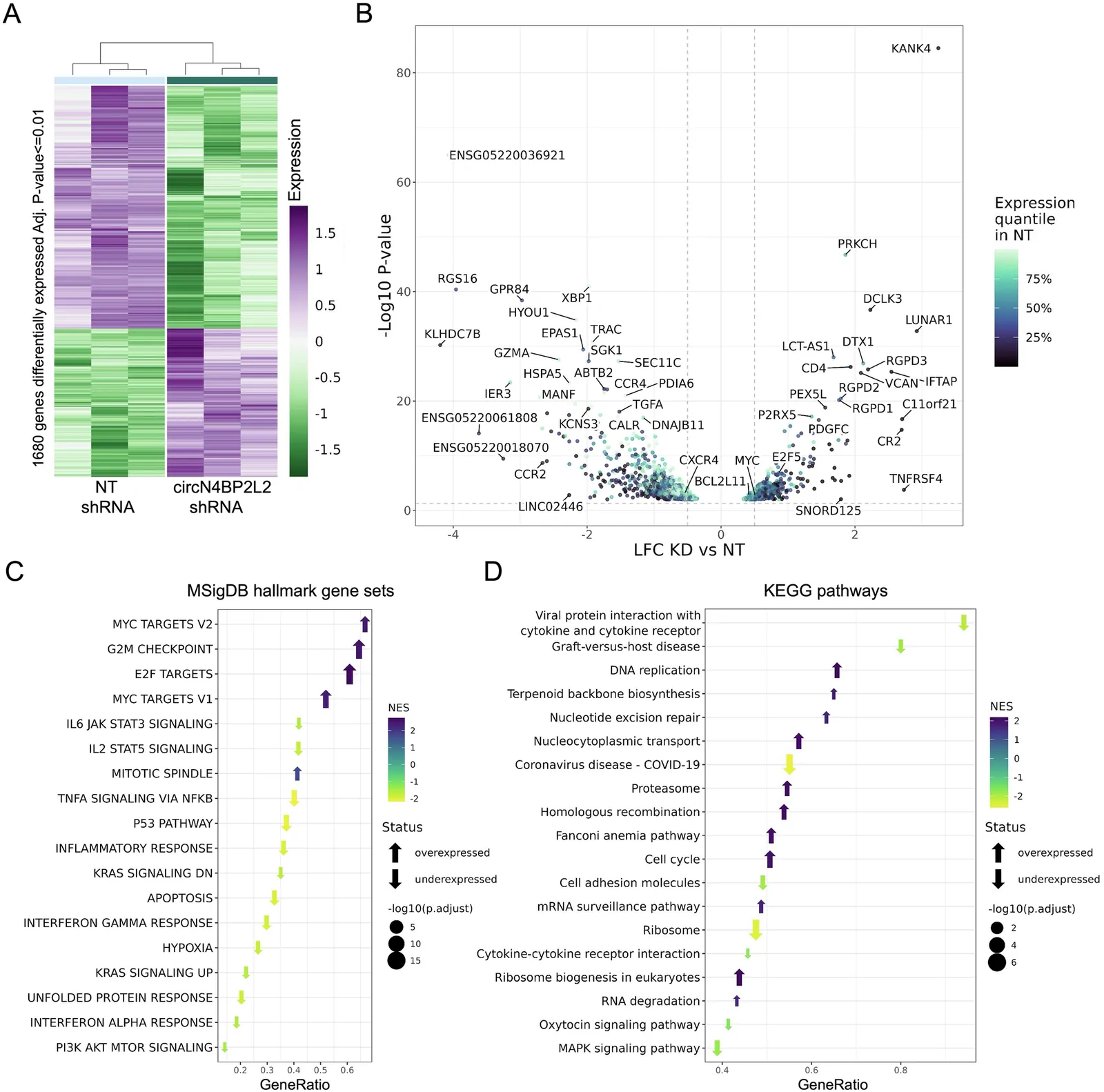
Impact of circN4BP2L2 silencing on gene expression profile in JURKAT cells. **A** Heatmap and **B** volcano plot of 1680 genes differentially expressed upon circN4BP2L2 silencing. C MSigDB Hallmark Gene sets and D The KEGG pathways significantly enriched upon circN4BP2L2 silencing according to GSEA analysis.

**Fig. 7:**
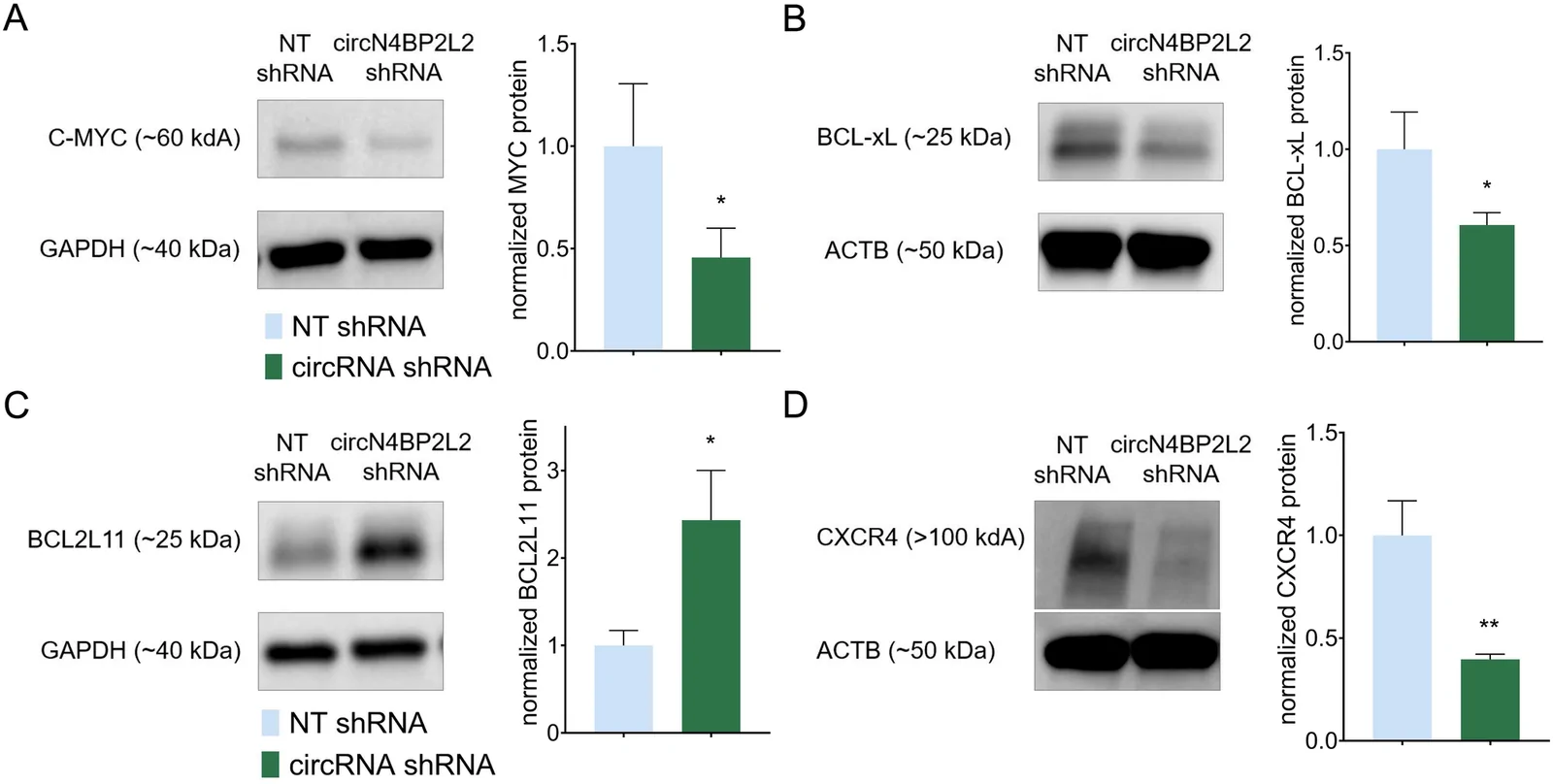
Impact of circN4BP2L2 silencing on the level of selected proteins in Jurkat cells. Relative quantification of c-MYC (**A**), BCL-xL (**B**), BCL2L11 (**C**) and CXCR4 (**D**) proteins in Western Blot upon repression of circN4BP2L2 in JURKAT cell line; * *p* < 0.05; ** *p* < 0.01.

To get insight into the impact of circN4BP2L2 on gene expression and pathway activation in T-ALL in vitro, we performed mRNA-seq in JURKAT cells with stable shRNA1-mediated repression of this circRNA compared to a non-targeting (NT) shRNA control. We observed a strong effect of circN4BP2L2 knockdown on the global transcriptomic landscape, with 1680 genes differentially expressed (635 up- and 1045 downregulated, with p-adj ≤0.01) (Fig. 6A, B). Gene Set Enrichment Analysis (GSEA) revealed several functional categories significantly enriched upon circN4BP2L2 silencing, including 26 MSigDB Hallmark Gene sets, 41 KEGG pathways, and 712 Gene Ontology Biological Functions (Supplementary Table S5). The most enriched and relevant gene sets and pathways are shown in Fig. 6C, D, and the top biological functions are shown in Supplementary Fig. S10. Interestingly, GSEA revealed enrichment of MYC and E2F targets, as well as genes involved in G2M checkpoint and mitotic spindle gene sets, among genes upregulated upon circN4BP2L2 silencing. We also noticed a significant increase in *E2F5* and *MYC* expression at the transcript level. In contrast to the *MYC* mRNA, MYC protein appeared significantly decreased upon circN4BP2L2 repression (Fig. 7A). A possible explanation, supported by the enrichment of circN4BP2L2 in the AGO2-IP fraction, is translation inhibition of *MYC* by miRNAs—no longer sequestered by circN4BP2L2 upon its silencing. MYC downregulation is consistent with the downregulation of genes involved in cytoplasmic translation and protein folding, revealed by GSEA. The increase in *MYC* transcript might, in turn, be explained by the dynamic autoregulatory ability of MYC [30]. We identified eight miRNAs expressed in T-ALL, predicted to bind circN4BP2L2 and having *MYC* as a validated target (Supplementary Fig. S11). These observations together suggest that repression of circN4BP2L2 disrupts the MYC-dependent gene expression networks, putatively in a miRNA-mediated manner.

Moreover, genes implicated in oncogenic pathways like TNFα signaling via NFκB, JAK-STAT, KRAS, PI3K-AKT, and stress-induced processes, such as the p53 pathway, apoptosis, and unfolded protein response, were enriched among those downregulated upon circN4BP2L2 silencing. Several genes linked to apoptosis regulation were modulated in a direction that supports the oncogenic role of circN4BP2L2. Of interest, *BCL2L1* and *IER3*, both encoding anti-apoptotic proteins, had decreased expression upon circN4BP2L2 silencing. In line, the level of BCL-xL, the anti-apoptotic protein encoded by *BCL2L1*, was decreased upon circRNA repression (Fig. 7B). Also, the *TNFRSF4* receptor, which promotes apoptosis inhibitors BCL2 and BCL2L1, was strongly downregulated. Instead, the proapoptotic *BCL2L11* expression was increased upon circN4BP2L2 silencing, and this was also confirmed by the BCL2L11 (alias BIM) protein level (Fig. 7C). Elevated BIM upon decreased MYC protein level is in agreement with previous reports on MYC-dependent repression of *BCL2L11/*BIM in T-ALL [31]. The perturbation of several apoptosis regulator genes could explain the increase of apoptosis upon circN4BP2L2 silencing (Fig. 5B). Upon repression of circN4BP2L2, we also observed a decrease in *CXCR4* expression, a pro-survival gene in T-ALL, that has previously been linked to this circRNA in colorectal cancer [32, 33]. This effect was further confirmed at the protein level (Fig. 7D).

Overall, circN4BP2L2 knockdown caused widespread gene deregulation, suggesting its involvement in networks essential for maintaining the oncogenic phenotype in T-ALL.

### T-ALL features related to high circN4BP2L2 expression overlap with those reversed by circN4BP2L2 silencing

**Fig. 8:**
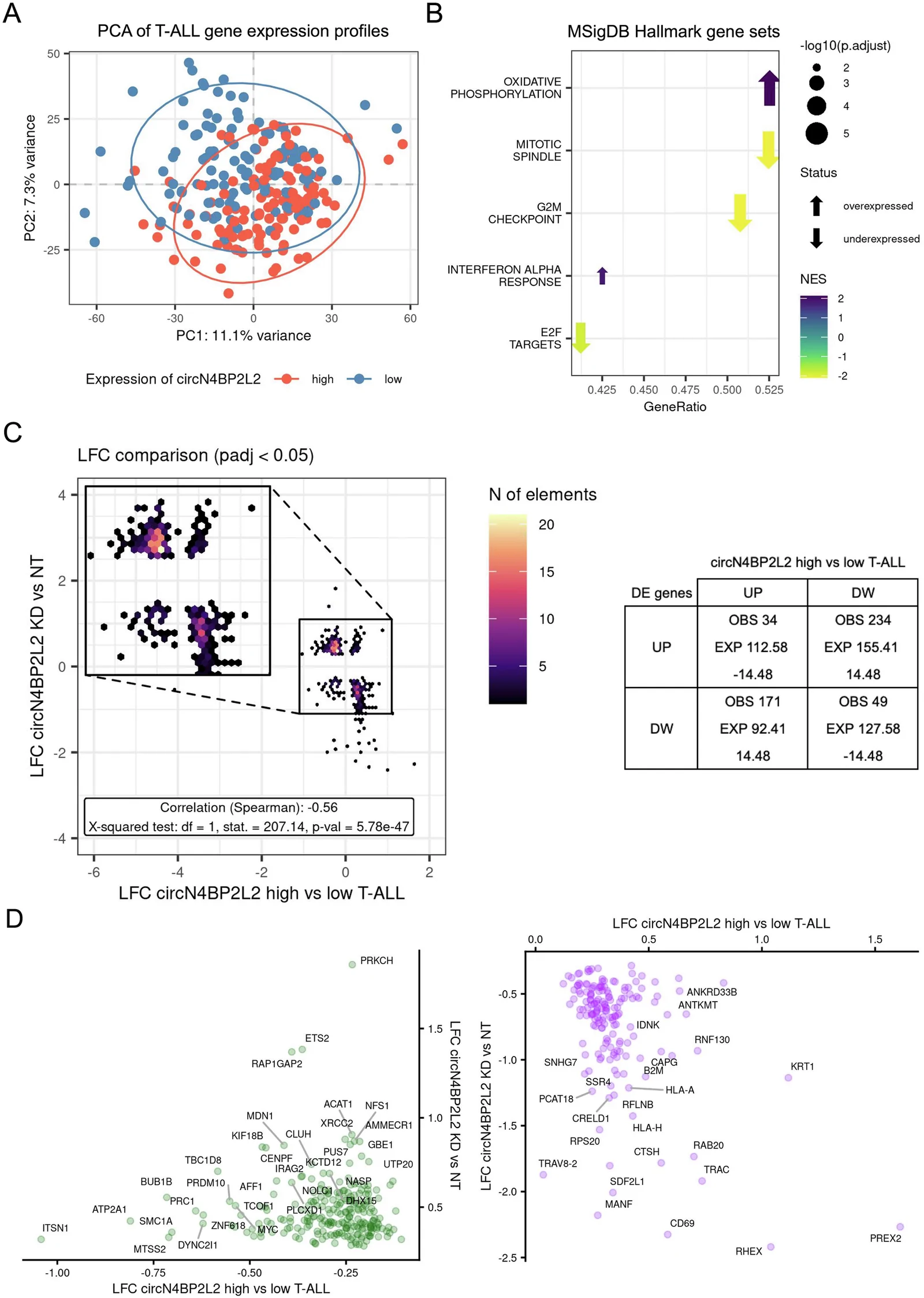
Gene expression differences in T-ALL patients stratified by circN4BP2L2 expression. **A** PCA of T-ALL samples according to gene expression profile (samples of circN4BP2L2 high and circN4BP2L2 low T-ALL are colored in red and blue). **B** The KEGG pathways significantly enriched in circN4BP2L2 high vs low T-ALL according to GSEA analysis. **C** Density contour plot of gene expression changes (LFC) observed upon circN4BP2L2 silencing in vitro and when comparing circN4BP2L2 high vs low T-ALL, and contingency table of the number of up- and down-regulated genes in the two comparisons indicating the Standardized Residuals comparing the observation (OBS) with the count expected under no association hypothesis (EXP), indicating a strong deviation from it. **D** Scatterplot of the gene expression changes (LFC) observed upon circN4BP2L2 silencing in vitro and when comparing circN4BP2L2 high vs low T-ALL for genes with opposite behavior in the two comparisons.

Finally, we quantified gene and circRNA expression of 242 pediatric patients with T-ALL. CircN4BP2L2 expression ranged in two orders of magnitude (normalized read count from 1.1 to 173.5, median 66.1). Samples were stratified according to circN4BP2L2 expression over (circN4BP2L2-high) or under (circN4BP2L2-low) the median of the cohort. PCA analysis of gene expression showed noticeable separation of samples of circN4BP2L2-high and -low T-ALL groups, indicating transcriptomic differences and possibly distinct cell states associated with circN4BP2L2 expression (Fig. 8A).

Direct comparison of circN4BP2L2-high and -low T-ALL patients revealed 2057 genes differentially expressed (p-adj <0.05), 1017 up- and 1740 down-regulated, linked to high circN4BP2L2 expression. The most upregulated included *LMO1*, *PREX2*, *TRGV8*, *PDLIM1*, *KRT1*, *RHEX*, whereas *MEIS1*, *ITSN1*, *MKI67*, *LUC7L3*, *SMC1A*, *TRIO* were among the most downregulated. GSEA revealed differential expression of genes involved in proliferative activity between circN4BP2L2-defined subgroups, and enrichment of ribosome and oxidative phosphorylation pathways among upregulated genes (Fig. 8B), which can result from oncogenic pathway activation. The upregulation of Interferon alpha response may contribute to apoptosis resistance and enhanced cell survival, as shown in other leukemias [34].

Interestingly, a strong anticorrelation (rho –0.56, *p* value 2.2^-16^) between expression variations observed in circN4BP2L2-high vs -low patients and upon circN4BP2L2 silencing vs control in vitro was observed (Fig. 8C). Indeed, 85.0% of the 488 common differentially expressed genes showed the opposite patterns of changes in the two comparisons, being more expressed in circN4BP2L2-high cases and downregulated upon circRNA silencing in vitro (171 genes), or vice-versa (243 genes), with a strong association (Cramer’s V 0.652, Chi-squared *p* value 5.78 × 10^−47^).

We considered that circN4BP2L2 expression could be associated with molecular subtypes of T-ALL. We therefore reanalysed the patients’ gene expression data correcting the gene expression profiles for the effect of the T-ALL molecular subtype. Main figures remained similar: 2757 differentially expressed genes comparing circN4BP2L2-high vs -low patients, and strong anticorrelation of the expression profiles with those observed upon circN4BP2L2 silencing in vitro (rho –0.51, *p* value 2.0^–16^). The integration of transcriptomic data upon circN4BP2L2 silencing in vitro and T-ALL patients allowed for the identification of genes positively (right panel) and negatively (left panel) associated with circN4BP2L2 expression (Fig. 8D). The robust concordance between the transcriptomic changes observed upon silencing in vitro and the expression patterns in patient samples strongly suggests that circN4BP2L2 may influence cellular pathways involved in sustaining the oncogenic phenotype in T-ALL.

Finally, to assess potential clinical relevance, we examined associations between circN4BP2L2 expression and patient outcomes. No significant differences were observed between patients with high versus low circN4BP2L2 expression in terms of survival status (alive/dead; Chi-squared test, *p* value 0.8), Overall Survival (OS; Cox proportional hazards, HR 1.33, *p* value 0.58), nor Event-Free Survival (EFS). These findings indicate that circN4BP2L2 expression does not significantly correlate with clinical outcome in this particular cohort.

## Discussion

Our study provides the first systematic interrogation of AGO2-associated circRNAs in T-ALL using AGO2-RIP-seq across multiple cell lines. By linking circRNA discovery to the RISC machinery in T-ALL, we identify a high-confidence subset of circRNAs with potential miRNA-mediated regulatory relevance, consistently across different T-ALL cell lines and after stringent bioinformatic filtering. In line with previous studies on cancer, we observed enrichment of a relatively small subset of circRNAs in RISC complexes in T-ALL. For these circRNAs, a potential role as miRNA decoys that relieve repression of oncogenic gene networks is suggested [35]. Our findings also indicate that other functions, beyond miRNA-related mechanisms, should be investigated for specific circRNAs. With these results, we provide novel insight into circRNAs that potentially govern miRNA-involving regulatory axes in T-ALL and present an extensive resource for further investigation of circRNA in hematologic malignancies.

While classic examples such as CDR1as demonstrate that circRNAs can function as efficient miRNA sponges [36], the general prevalence of this mechanism remains debated. Only a minority of circRNAs harbor multiple binding sites for a single miRNA, making widespread potent sponging unlikely [37]. Other studies showed that even circRNAs with one or two binding sites can significantly modulate miRNA activity when expressed abundantly [38]. Since the “sponging” effect is based on a ceRNA mechanism, such circRNA must be abundant enough to effectively sequester a significant number of miRNA molecules. Moreover, a single circRNA may simultaneously bind multiple miRNAs that converge on the same regulatory network, thereby amplifying its ceRNA effect [39]. The impact of a circRNA on miRNA activity depends not only on the number of miRNA response elements but also on the balance between circRNA and miRNA levels: abundant miRNAs are minimally affected by individual response elements, whereas low-abundance miRNAs can be strongly influenced by highly expressed circRNAs [40]. According to our AGO2-RIP-seq data from T-ALL cell lines, an average of 7.3% of expressed circRNAs were enriched in the AGO2-IP fractions, with several showing high expression levels. These circRNAs are therefore candidates for miRNA sponge activity. Integration with previously published RNA-seq profiles from T-ALL patient samples and normal thymocytes [21] enabled us to identify a panel of RISC-associated circRNAs that are dysregulated, abundant, and may contribute to leukemogenesis in T-ALL.

Our main highlight is the discovery of the oncogenic role of circN4BP2L2 in T-ALL in vitro. We validated the circular structure and backsplice junctions of eight circRNAs concordantly enriched in the AGO2-IP fractions in several cell lines, upregulated in T-ALL samples compared to the normal counterpart, and highly expressed in leukemic cells, highlighting them as candidates for functional follow-up. Of note, none of these circRNAs originates from transcripts with known roles in T-ALL. This phenomenon was also observed in other malignancies [41, 42] and underscores the discovery power of studies focused on circRNAs.

After an initial functional screening, neither circZNF124 nor circGAPVD1 repression resulted in marked changes in survival of T-ALL cell lines. CircZNF124 has been previously described as potentially oncogenic in endometrial and non-small cell lung cancers [43], however our RNA-seq data indicate the presence of several circRNAs with partially overlapping sequences, which might compensate for the effects of the knockdown. In our competition assay, the repression of circGAPVD1 previously shown to inhibit gastric cancer progression [44] resulted in a very modest decrease in survival advantage, only after a long culture period and only in one cell line. Notably, concordant results in three different T-ALL cell lines indicated circN4BP2L2 as a strong candidate with potential oncogenic properties. This circRNA has been previously reported as oncogenic in colorectal cancer [33, 45] and non-small cell lung cancer [46] and as a promising biomarker in ovarian cancer [47]. With further experiments, we clarified its cytoplasmic location. Functional assays using two shRNAs revealed that circN4BP2L2 promotes proliferation and confers resistance to apoptosis in T-ALL cell lines.

By transcriptomic profiling following circN4BP2L2 knockdown in vitro, we uncovered profound alterations in gene expression, notably affecting pathways central to T-ALL pathogenesis. We confirmed, both at the transcript and protein level, the effect of circN4BP2L2 on the increased expression of oncogenic *CXCR4* previously reported as protected by this circRNA from miR-340-5p repression via the ceRNA mechanism in colorectal cancer [33]. We also revealed its ambiguous influence on MYC signaling. The observed discrepancy between increased *MYC* mRNA and reduced MYC protein upon circN4BP2L2 silencing, along with several miRNAs targeting *MYC* predicted to bind to circN4BP2L2, supports our hypothesis that this circRNA is involved in MYC-related oncogenic networks in this leukemia, possibly by simultaneously sequestering multiple *MYC*-targeting miRNAs. The downregulation of MYC protein upon circN4BP2L2 silencing is in line with the functional effects observed in vitro: reduced cell viability and enhanced apoptosis. This might reflect the shift from pro-survival towards pro-apoptotic pathways, leaving the cells in a vulnerable state.

Surprisingly, despite the decrease in MYC protein upon circN4BP2L2 silencing, GSEA revealed upregulation of several genes assigned as MYC targets. This discrepancy might be explained by several factors. MYC directly or indirectly controls 10–15% of genes in the human genome with both activating and repressing impacts. MYC effect can be context-dependent, varying by tissue type, cell state, and cofactor availability [48, 49]. In fact, upon circN4BP2L2 silencing, several genes previously described as repressed by MYC in T-ALL (*BCL1L11*, *HES4*, *PTCRA*, *IL7R*, *DTX1*) [31, 50] were found to be increased, which is expected when the MYC level is decreased. Furthermore, upon the reduced level of MYC protein, compensatory mechanisms aiming to restore the expression of its targets (e.g., activity of other transcription factors) might be activated. Notably, we postulate that circN4BP2L2 is involved in posttranscriptional regulation via miRNA binding and thus some effects of its knockdown may not be detectable at the transcript level [51]. Moreover, gene expression regulation is a dynamic process, strongly context-dependent, and our transcriptome analysis performed at a precise time point could not fully capture the temporal dynamics.

Finally, by analysis of a sizable T-ALL cohort, we showed that T-ALL patients are heterogeneous in terms of circN4BP2L2 expression and can be divided into two subgroups, with high or low circN4BP2L2 abundance. No significant association was observed between circN4BP2L2 expression and clinical outcomes. This might be attributed to the limited number of relapses and adverse events in this cohort, reducing the power to detect such associations. Nonetheless, this does not diminish the biological relevance of our findings. These groups are characterized by a different transcriptomic profile, even if controlling for the effect of genetic subtypes. We show that knockdown of circN4BP2L2 in vitro impacts the gene expression landscape, making it more similar to the one observed in circN4BP2L2-low patients. Among genes more expressed in circN4BP2L2-high T-ALL and significantly downregulated upon circN4BP2L2 silencing, several play oncogenic roles, e.g., *CXCR4, PTP4A3, GPX4, PREX2, DDIT4, CAPN1, RHEX, PTPA3, SOX3* and *EEF1D* [32, 52–55]. On the other hand, among genes less expressed in circN4BP2L2-high T-ALL and significantly upregulated upon circN4BP2L2 silencing, there are several tumor suppressors, e.g., *FANCD2, FANCA, MSH6, XRCC2, GEN1, FNIP2*, and *BCL2L11* [56–58]. These observations in patient samples further corroborate our in vitro functional data and support the postulated role of circN4BP2L2 in shaping and maintaining the oncogenic phenotype in a subgroup of T-ALL cases. As a future direction, in vivo xenograft studies using circN4BP2L2-silenced T-ALL cells, complemented with functional and mechanistic analyses will be essential to more precisely define the potential oncogenic role of circN4BP2L2 in T-ALL pathobiology.

### Data Sharing Statement

RNA-seq data are freely available at ArrayExpress (https://www.ebi.ac.uk/biostudies/arrayexpress/) under the accession numbers E-MTAB-16337 (AGO2-RIP-seq data) and E-MTAB-16358 (mRNA-seq).

## Supplementary information

Supplementary Methods and Results

Supplementary Table 4

Supplementary Table 5

## Data Availability

The RNA-seq data produced in this study are available under the accession numbers E-MTAB-16337 and E-MTAB-16358 at the ArrayExpress database.

## Code availability

Code used for data analysis is available from the corresponding author upon request.
