# Supplementary material for: Systematic profiling of AGO2-associated circRNAs and functional investigation reveal circN4BP2L2 as a survival-promoting regulator in T-ALL
Source: Leukemia. 2026 Jul 27;40(10):2223–35. doi: 10.1038/s41375-026-03033-x (PMC13612239; doi:10.1038/s41375-026-03033-x)
Supplement: Supplementary file 1 [file 41375_2026_3033_MOESM1_ESM.pdf]

Systematic Profiling of AGO2-associated circRNAs and Functional Investigation Reveal circN4BP2L2 as a Survival-promoting Regulator in T-ALL

Monika Drobna-Sledzinska[1]*, Alessia Buratin[2,3]*, Eleonora Roncaglia[4,3], Alberto Caregari[4,3], Ilias Glogovitis[2], Maria Kosmalska[1], Xing Zhao[5], Silvia Bresolin[6], Enrico Gaffo[4], Anke Van den Berg[5], Joost Kluiver[5], Malgorzata Dawidowska[1$], Stefania Bortoluzzi[4$&].

1 Institute of Human Genetics, Polish Academy of Sciences, Poznan, Poland.

2 Department of Molecular Medicine, University of Padova, Padova, Italy.

3 Department of Biology, University of Padova, Padova, Italy.

4 Department of Oncology Surgery and Gastroenterology, University of Padova, Padova, Italy.

5 Department of Pathology and Medical Biology, University of Groningen, University Medical Centre Groningen, Groningen, the Netherlands.

6 Onco-Hematology, Stem Cell Transplant and Gene Therapy Laboratory, IRP-Istituto Di Ricerca Pediatrica, Padua, Italy.

*These authors contributed equally

$ Co-last authors

& Corresponding

Supplementary Information

Supplementary Methods 2

Culture of Cell Lines 2

RNA Extraction 2

miRNA RT-qPCR 2

RNA Sequencing 2

Detection and quantification of circRNAs and linear transcripts 3

Enrichment score calculation 3
CircRNA sequence confirmation by RT-PCR and Sanger sequencing 4
Subcellular fractionation 5
Immunoblotting 5
Lentiviral transduction 5
GFP competition assay 6
Proliferation Assay 6
Apoptosis Assay 6
Gene expression analysis upon circN4BP2L2 silencing 6
Supplementary Figures 8
Supplementary Figure S1. CircRNAs detection in the AGO2-IP and TOTAL fraction of each cell line. 8
Supplementary Figure S2. Number of linear transcripts detected in the AGO2-IP and TOTAL fraction of each cell line. 9
Supplementary Figure S3. Saturation curves for the different T-ALL cell lines sequenced in AGO2-IP (left) and TOTAL (right) RNA fraction. 9
Supplementary Figure S4. Density plot of circRNA abundance (BJR, backsplice junction read count) in the AGO2-IP and TOTAL fractions of the different T-ALL cell lines. 11
Supplementary Figure S5. Fraction of linear transcripts per gfold comparing AGO2-IP fraction vs TOTAL fraction in the four T-ALL cell lines. 12
Supplementary Figure S6. Comparison of AGO2-fraction enrichment for circular and linear RNAs in four T-ALL cell lines. 13
Supplementary Figure S7. Validation of circRNAs. 14
Supplementary Figure S8. Quantification of circN4BP2L2 in the nuclear and cytoplasmic RNA fractions of T-ALL cell lines. 15
Supplementary Figure S9. Exemplary flow cytometry histograms from Annexin-V APC apoptosis assay upon shRNA-mediated circN4BP2L2 knockdown in JURKAT and DND-41 T-ALL cell lines 16
Supplementary Figure S10. Gene Ontology Biological Processes that were significantly enriched upon circN4BP2L2 silencing according to GSEA analysis. 17
Supplementary Figure S11. CircN4BP2L2 presents predicted binding sites for different miRNAs targeting MYC. 18
Supplementary Tables 19
Supplementary Table S1. List of primers used for RT-qPCR detection of studied circRNAs and linear transcripts. 19
Supplementary Table S2. List of primers used for RT-PCR and Sanger sequencing of selected circRNAs. 20
Supplementary Table S3. List of oligonucleotides used for cloning into shRNA expression vectors. 20
Supplementary Table S4. List of 144 circRNAs enriched in AGO2-IP in at least three T-ALL cell lines. 22
Supplementary Table S5. List of enriched terms upon circN4BP2L2 silencing in JURKAT cells according to GSEA. 22
References 22

Supplementary Methods

Culture of Cell Lines

DND-41, ALL-SIL and LOUCY cell lines were purchased from DSMZ (Brunswick, Germany). JURKAT cell line was a kind gift from Dr. Katarzyna Iżykowska (Institute of Human Genetics, Polish Academy of Sciences). Cells were cultured under standard conditions in RPMI-1640 medium (Thermo Fisher Scientific, Waltham, MA, USA) with 10% (JURKAT, LOUCY, DND-41) or 20% (ALL-SIL) of fetal bovine serum (Thermo Fisher Scientific).

RNA Extraction

The miRNeasy micro RNA isolation kit (Qiagen, Hilden, Germany) was used for the extraction of total RNA (TOT) and AGO2-IP flow-through RNA. RNA isolates were DNase-treated and purified with the use of RNA Clean and Concentrator Kit (Zymo Research, Irvine, CA, USA). AGO2-IP RNA was isolated directly using the RNA Clean and Concentrator Kit. RNA concentration was measured with Quantus Fluorometer (Promega, Madison, WI, USA) using Qubit HS RNA Assay Kit (Thermo Fisher Scientific). RNA integrity was determined with 4200 Tapestation using High Sensitivity RNA ScreenTape (Agilent Technologies, Santa Clara, CA, USA).

miRNA RT-qPCR

For miRNA quantification, RNA was reverse transcribed with TaqMan Advanced miRNA cDNA Synthesis Kit (Thermo Fisher Scientific) according to the manufacturer's protocol. TaqMan Fast Advanced Master Mix and predesigned TaqMan Advanced miRNA assays (Thermo Fisher Scientific, assays 477860_mir, 478575_mir and 477994_mir) were used. Fold enrichment of examined miRNAs in AGO2-IP fraction was calculated as $2^{-\Delta Cq}$ (Cq—quantification cycle), where $\Delta Cq = Cq(AGO2\text{-}IP) - Cq(IgG\text{-}IP)$.

RNA Sequencing

Libraries were constructed from the total (TOT) and AGO2-IP RNA fraction samples in the 4 T-ALL cell lines, using TruSeq Stranded Total RNA with Ribo-Zero Human/Mouse/Rat protocol, based on ribo-depletion. Fragmentation was performed at 90′C for 2 minutes, as described previously [1]. The libraries were sequenced on an Illumina NovaSeq6000 platform,

using the following settings: 2x150 PE (paired end sequencing with 150 nt reads), read depth of coverage: 150 million read pairs/sample.

For gene expression profiling in Jurkat cell line upon repression of circN4BP2L2, libraries were constructed using TruSeq Stranded mRNA kit and were sequenced on Illumina NovaSeqX platform (2x150 PE, 150 million read pairs/sample).

Detection and quantification of circRNAs and linear transcripts

The RNA-seq data have been analyzed with the CirComPara2 bioinformatics pipeline[2] to identify and quantify back-spliced junction (BSJ) read counts. CircRNA annotation and all analyses were based on the Ensembl GRCh38 human genome and annotation v97. CircRNAs reported by at least two methods with at least two reads were considered reliable. CircRNA counts were normalized to the total number of uniquely aligned reads to obtain counts per million (CPM). Moreover, non-circular reads aligned across the BSJ sites were used to quantify the linear counterpart and calculate the circular-to-linear proportion (CLP) as in Buratin *et al*.[3]. In more detail, to compare circRNA abundance with their cognate linear transcripts, for each backsplice junction, we calculated the ratio between the number of backspliced reads and the reads linearly spliced on the same junctions defining the backsplice. Unspliced reads aligned to exon bodies were excluded, as they may derive from either the circRNA or its linear cognate[2,4].

For linear transcript expression analysis, standard protocols with HISAT2[5] and StringTie[6] were implemented. In brief, the sequencing reads were aligned to the reference genome using HISAT2, and StringTie was used to quantify transcript expression levels. The linear transcript read counts were normalized to the total number of uniquely aligned reads to obtain counts per million (CPM). Enrichment score calculation was performed as described below for circRNA analysis.

To assess the saturation level of RNA-seq samples (i.e., the adequacy of the sequencing depth to assure adequate statistical power), we used the subsampling methodology NOISeq[7]. We randomly subsampled the raw reads to different sequencing depths (from 10% to 90%, at intervals of 10%) and measured their performance in terms of the fraction of detected circRNAs and linear transcripts.

Enrichment score calculation

The generalized fold change (gfold)[8] of the observed BSJ reads number was used as a measure of circRNA abundance difference between AGO2-IP and TOT RNA fractions. In

brief, the BSJ read count of a gene can be modeled by the Poisson distribution, so the probability of observing k BSJ reads from a circRNA can be defined as:

$$P(k) = \frac{\lambda^k e^{-\lambda}}{k!}, \lambda = n \times x \qquad (1)$$

where λ is the expression level of circRNA, measured by CPM, and n is a normalization factor of sequencing depth. In the Bayesian view, the posterior distribution of λ is defined by:

$$\text{Post}(\lambda) \propto \frac{\lambda^k e^{-k}}{k!} \qquad (2)$$

which is a gamma distribution with shape k+1 and scale 1. Thus, under two different conditions, the posterior distribution of expression levels x_1 and x_2 and the log2 fold change $z = \log_2(x_2/x_1)$ can be effectively calculated. Then, the enrichment score can be calculated by gfold, utilizing the variance information of the posterior distribution of fold change, Z:

$$\mathbf{gfold}(\mathbf{c}) = \begin{cases} max(t,0)|P(z \leqslant t) = c \quad if \quad mean(Z) \geqslant 0 \\ min(t,0)|P(z \geqslant t) = c \quad if \quad mean(Z) < 0 \end{cases} \qquad (4)$$

where P is the posterior distribution of $z = \log_2(x_2/x_1)$ and c is a parameter with default parameter 0.05. If the transcript is up-regulated, i.e., $mean(Z) \geq 0$, the probability of the $\log_2$ fold change being larger than t is $1-c$. In this case, t is less than $mean(Z)$ with a default value of c. *gfold*(c) takes $max(t,0)$ to truncate negative t to zero. If $gfold(c)$ is 0, then the expression level of this transcript does not show a significant change. The case for $mean(Z) \leq 0$ is symmetric to the case of $mean(Z) \geq 0$. Transcripts can be ranked by their gfold values in descending order, such that top-ranked ones are differentially up-regulated and bottom-ranked ones are differentially down-regulated. The output gfold balances the fold change and p-value methods, which provides an effective way to rank enriched transcripts.

CircRNA sequence confirmation by RT-PCR and Sanger sequencing

To determine the full sequences of selected circRNAs identified based on back-splice junction (BSJ) reads in total RNA-seq data, Sanger sequencing was employed. Primers for RT-PCR were specifically designed to span the BSJ, ensuring amplification of the circular RNA. Each circRNA was sequenced in overlapping fragments, using cDNA from T-ALL cell lines as a template. The primers used for RT-PCR are listed in **Supplementary Table 2**.

Subcellular fractionation

Subcellular fractionation of cellular and nuclear fractions was performed as described previously[9]. RNA from the fractions was isolated, reverse transcribed and used for qRT- PCR as described above. All samples were tested for circN4BP2L2 as well as *GAPDH*, *ACTB* (cytoplasmic controls), and *U3SNORNA* (nuclear control). The sum of the cytoplasmic and nuclear expression levels of each transcript was set to 100%, and the percentage of each transcript localized to each compartment was determined.

Immunoblotting

Proteins were separated on 4%–15% Mini-PROTEAN TGX Stain-free Gel (Bio Rad, Hercules, CA, USA). After electrophoresis, proteins were transferred onto a 0.45 µm PVDF Low Fluorescence membrane (Bio Rad). Membranes were blocked using 5% non-fat milk and incubated with 1:1000 anti-AGO2 (Ab32381, Abcam, Cambridge, UK), 1:2000 anti-MYC (Ab32072, Abcam), 1:2000 anti-BIM (#2933, Cell Signaling Technology, Danvers, MA, USA), 1:1500 anti-BCL-xL (Ab32370, Abcam), 1:1500 anti-CXCR4 (Ab124824, Acbam), 1:5000 anti-ACTB (#4970, Cell Signaling Technology) or 1:10000 anti-GAPDH (14C10, Cell Signaling Technology). After washing, membranes were incubated with horseradish peroxidase (HRP) conjugated with 1:40000 anti-rabbit secondary antibodies (Ab97051, Abcam). Immunoreactive protein bands were detected with Clarity Western ECL Substrate for HRP (Bio-Rad) on Chemidoc Imaging System (Bio-Rad). CXCR4 was detected predominantly as a high–molecular weight species, consistent with post-translationally modified and oligomeric forms reported for this protein.

Lentiviral transduction

For assembly of lentiviral particles, HEK293T cells were seeded and transfected as described previously[10]. For transduction of T-ALL cell lines, cells were seeded and then filtered medium containing lentiviral particles was added to each well. Polibrene Reagent (Sigma Aldrich) was added to each well at a final concentration of 8 µg/ml. Cells were spinfected for 90 min (at 1500 rpm and 32°C). Antibiotic selection of transduced cells was started 3–4 days post-transduction. For selection, puromycin dihydrochloride (Gibco, Thermo Fisher Scientific) was used at a concentration of 10 µg/ml for 4 days. The effectiveness of transduction was assessed with the use of the CytoFlex S flow cytometer (Beckman Coulter) with GFP as a marker.

GFP competition assay

For the GFP competition assay, cell lines were transduced with miRZip NT vector or miRZip circRNA shRNA vector with an efficiency of 20–80% without puromycin selection. Three days post-transduction, half of the cells from each well were harvested and the percentage of transduced (GFP+) cells was assessed using a CytoFlex S flow cytometer (Beckman Coulter). Cells were harvested every 2-3 days for 18-21 days, and the percentage of GFP+ cells for each well was calculated as a fold change of the percentage of GFP+ cells in the first tested time point. The experiment was performed in three independent biological replicates. The significance of the difference in normalized GFP+ rate in the analyzed time points between variants was tested with two-way ANOVA.

Proliferation Assay

Cultured cells were collected 4 days post-transduction and seeded onto 96-well plates. Cell Counting Kit-8 (Merck) reagent was added to the plates 0, 24, 48 and 72 hours after seeding. Cells were incubated with CCK8 reagent for 3 h at 37 °C. Absorbance was measured on the GloMax-Multi+ Detection System (Promega). The experiment was conducted in three technical and three biological replicates. The significance of the difference in proliferation rate between variants was tested with two-way ANOVA.

Apoptosis Assay

Cultured cells were collected 5 days post-transduction and stained with Annexin V APC (BD Biosciences, San Jose, CA, USA). Populations of apoptotic and non-apoptotic cells were detected with the Cytoflex S flow cytometer (Beckman Coulter, Brea, CA, USA). The experiment was conducted in three biological replicates. The mean percentages of apoptotic cells obtained for each variant were tested for normal distribution with the Shapiro-Wilk test, and the significance of the difference between the means was evaluated with an unpaired two-tailed t-test.

Gene expression analysis upon circN4BP2L2 silencing

Gene expression was quantified from mRNA-seq data generated upon silencing of circN4BP2L2 in cultured cells using the CircComPara2 [2] pipeline. Analysis focused on 12,637 genes (out of 61,951 detected) with TPM ≥ 1 in at least three samples, or in at least two per group. Differential gene expression analysis was conducted using DESeq2 v1.46.0 [11] with an adjusted p-value threshold ≤ 0.01 [11]. Wald tests were used for significance testing, and

p-values were adjusted for multiple testing using the Benjamini–Hochberg method. Gene set enrichment analysis (GSEA) was performed using clusterProfiler v4.14.4 [12] and enrichplot v1.26.6 [13]. All the analyses were carried out in the R v4.4.2 environment.

To build up the regulatory network involving circN4BP2L2, we considered 588 human miRNAs expressed in T-ALL according to small RNA-seq data (> 5 reads on average in T-ALL patients; Wallaert et al. 2017). Predictions of miRNA binding sites in the circN4BP2L2 sequence were obtained using four tools (MiRanda, TargetScan, IntaRNA and PITA), keeping only high confidence predictions [14] by applying cutoffs on free energy (ΔG, in kcal/mol; ≤ -20 kcal/mol for miRanda, ≤ -10 kcal/mol for PITA, and ≤ -10 kcal/mol for IntaRNA) and discarding small (6mer) sites predicted by TargetScan and miRanda with an alignment score lower than 140.

Supplementary Figures

Supplementary Figure S1. CircRNAs detection in the AGO2-IP and TOTAL fraction of each cell line.

A) Number of circRNAs detected in the AGO2-IP and TOTAL fraction of each cell line (the grey color indicates the number of circRNAs commonly detected in both fractions); (B) Multidimensional Scaling plot of sample distance based on circRNA expression profiles.

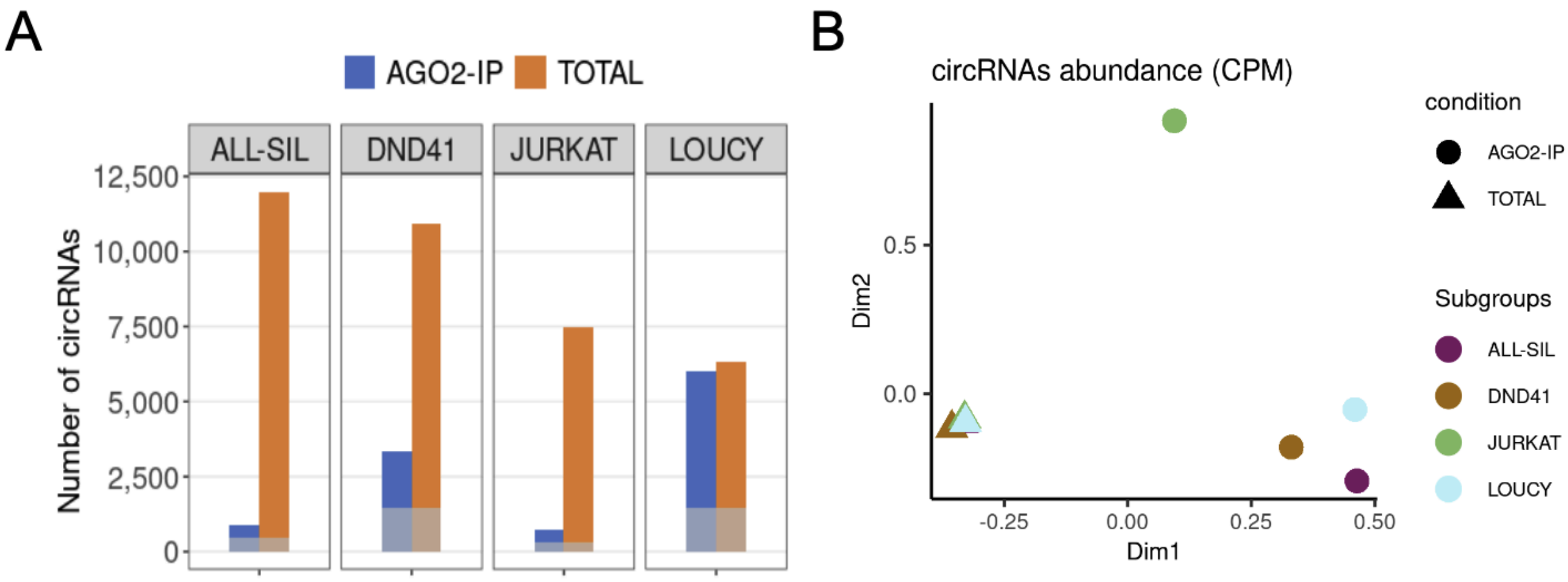

Supplementary Figure S2. Number of linear transcripts detected in the AGO2-IP and TOTAL fraction of each cell line.

The grey color indicates the number of transcripts commonly detected in both fractions.

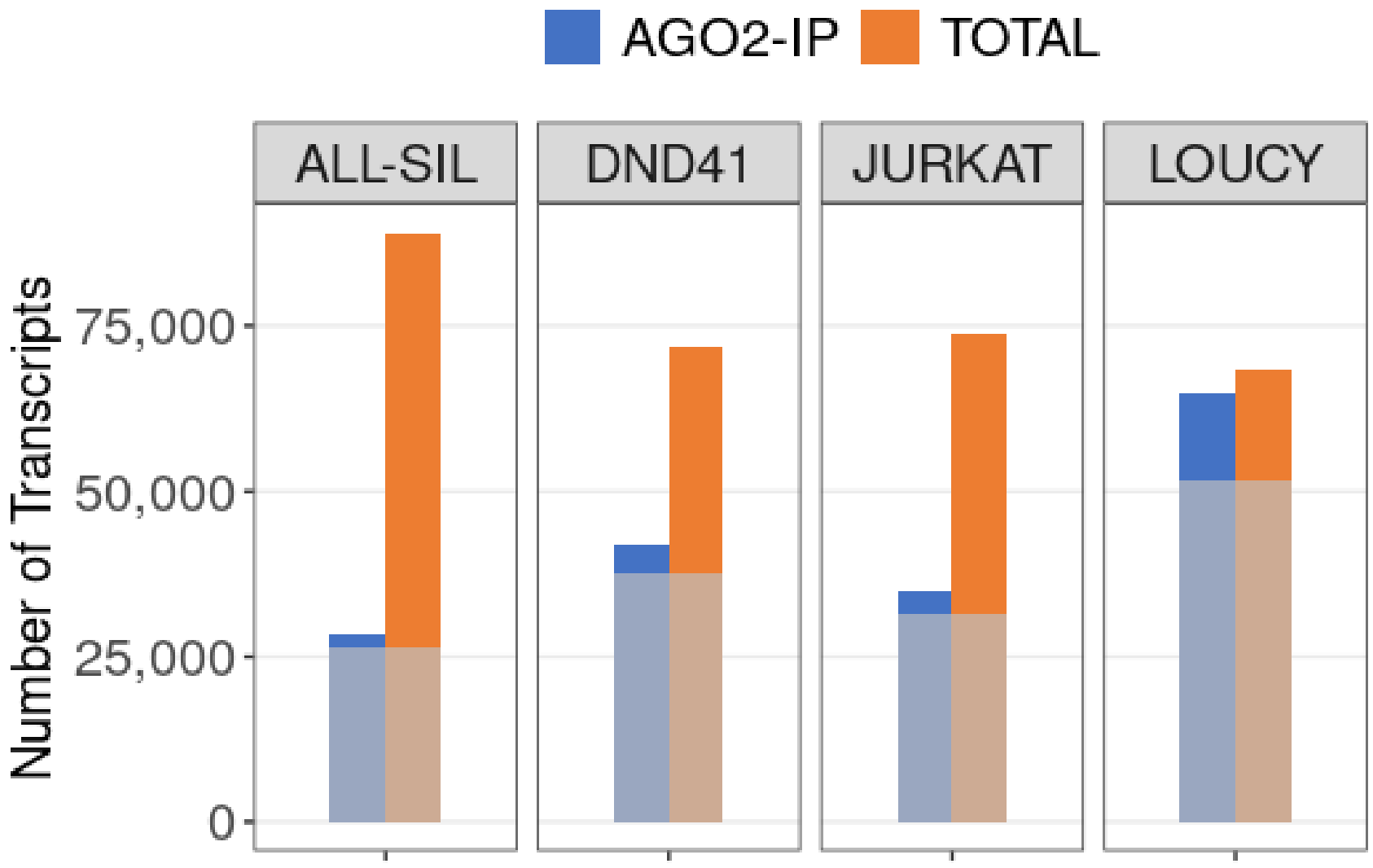

Supplementary Figure S3. Saturation curves for the different T-ALL cell lines sequenced in AGO2-IP (left) and TOTAL (right) RNA fraction.

The number of reads sequenced is on the x-axis, and the number of circular transcripts detected is on the y-axis. Solid dots indicate the real library size and the corresponding number of circRNAs detected. Each empty dot on the curve was generated by randomly decreasing or increasing the raw read counts from each sample library.

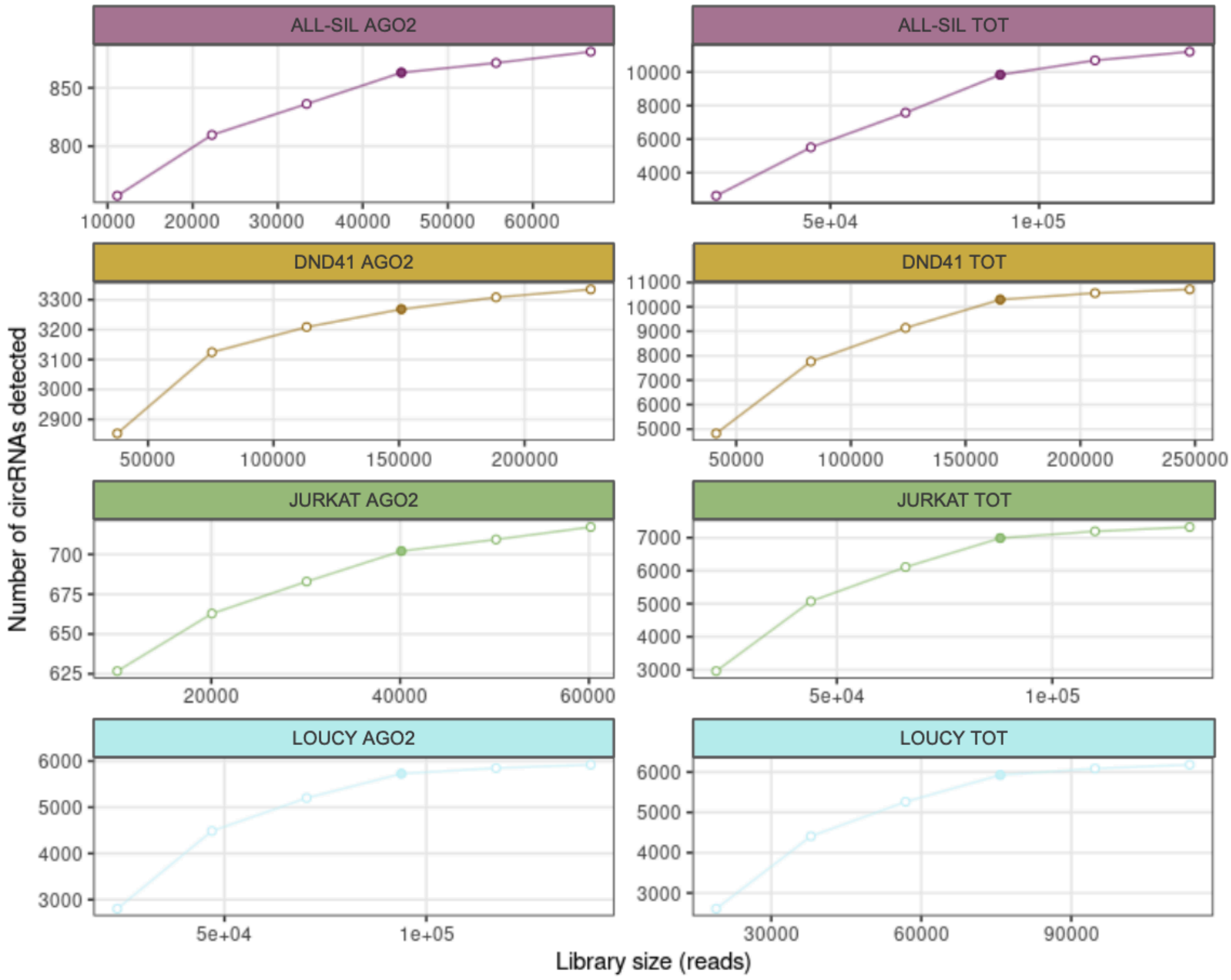

Supplementary Figure S4. Density plot of circRNA abundance (BJR, backsplice junction read count) in the AGO2-IP and TOTAL fractions of the different T-ALL cell lines.

The density peaks are more shifted towards higher values in the AGO2-IP fraction indicating that circRNAs abundance was higher in AGO2-IP than in the TOT fraction.

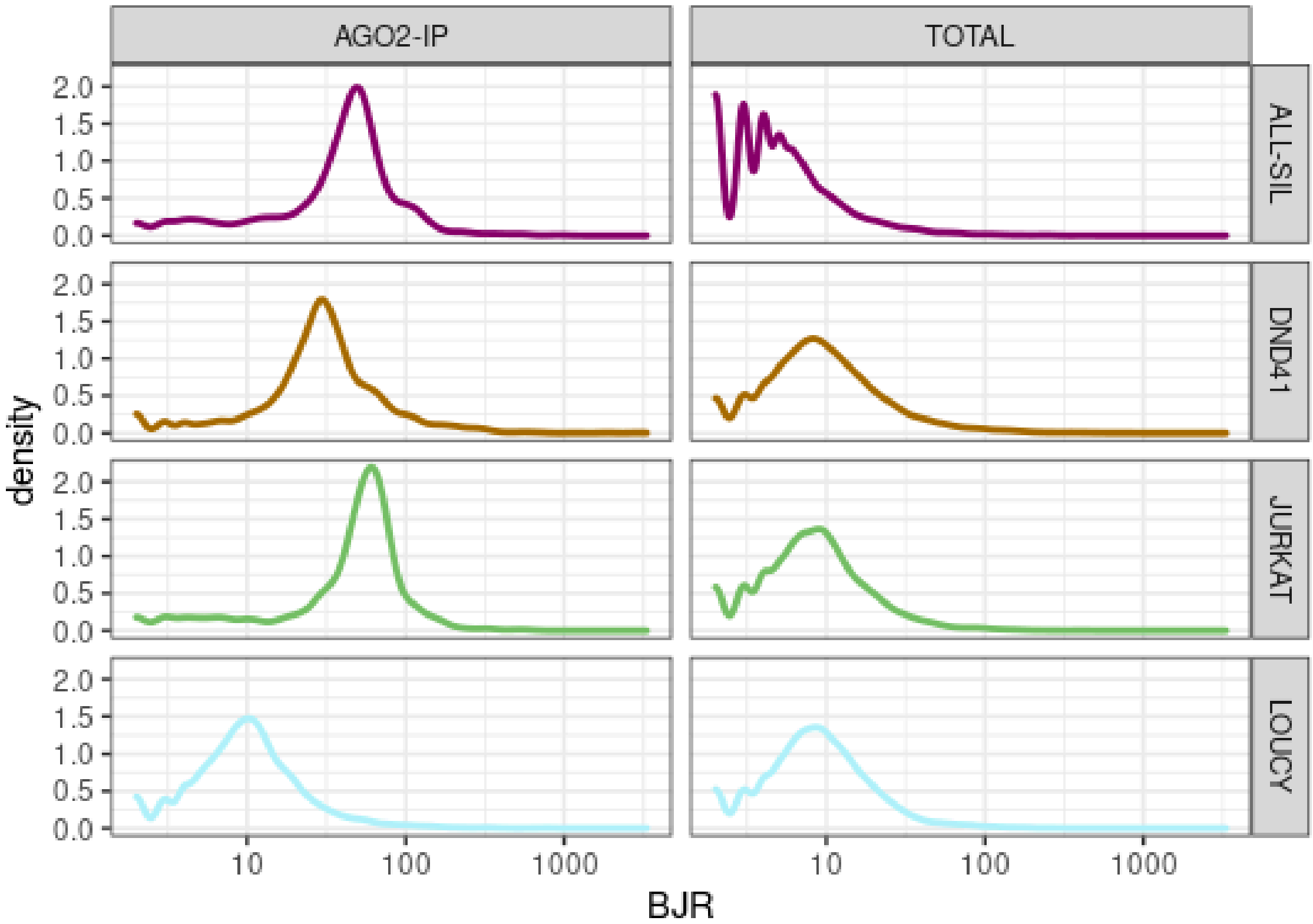

Supplementary Figure S5. Fraction of linear transcripts per gfold comparing AGO2-IP fraction vs TOTAL fraction in the four T-ALL cell lines.

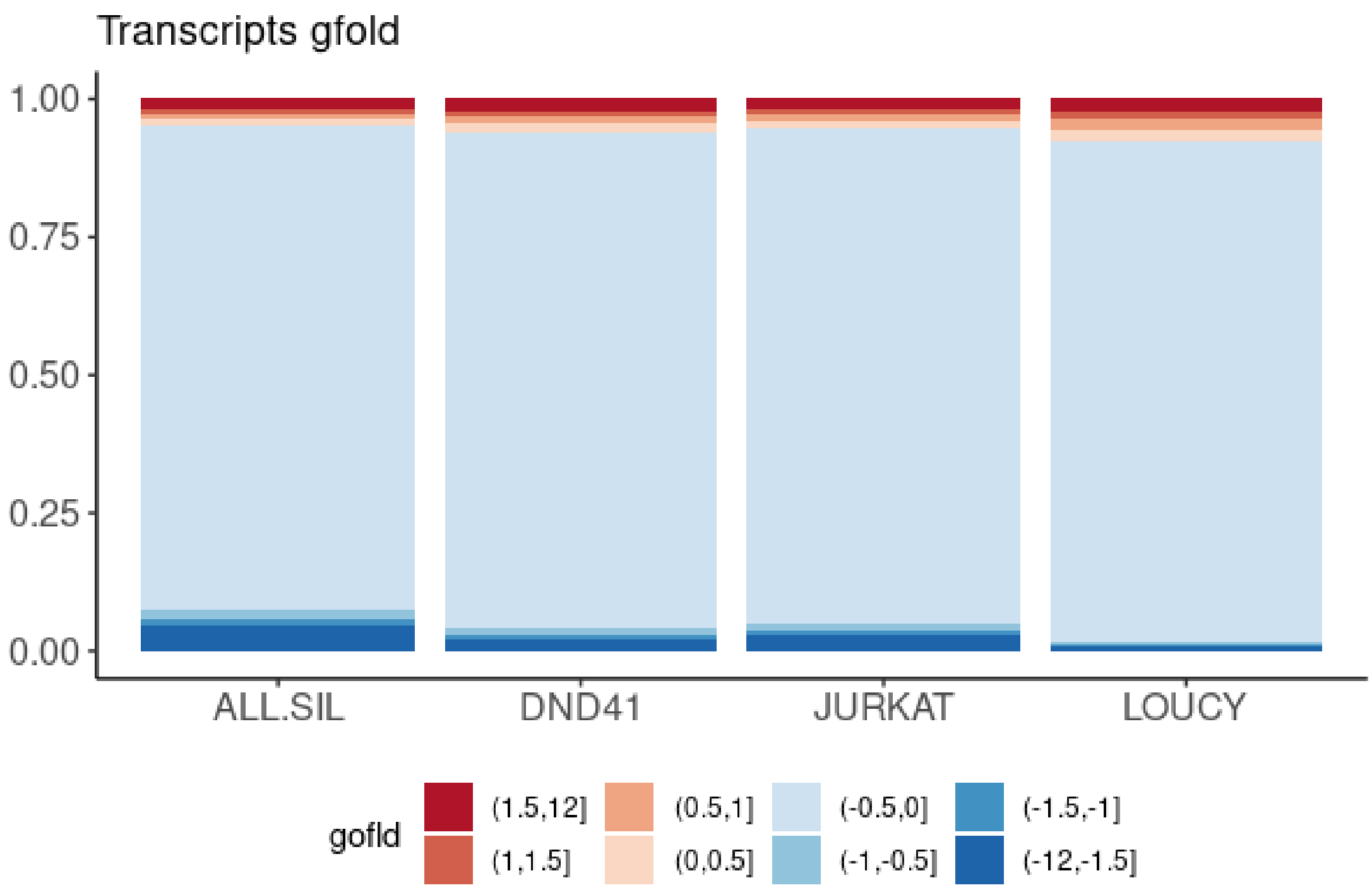

Supplementary Figure S6. Comparison of AGO2-fraction enrichment for circular and linear RNAs in four T-ALL cell lines.

The density distribution of circular (red) and linear (blue) transcripts enrichment (gfold comparing AGO2-IP vs TOTAL fraction) is shown, for each cell line; the dotted line shows the threshold used to define enriched RNAs.

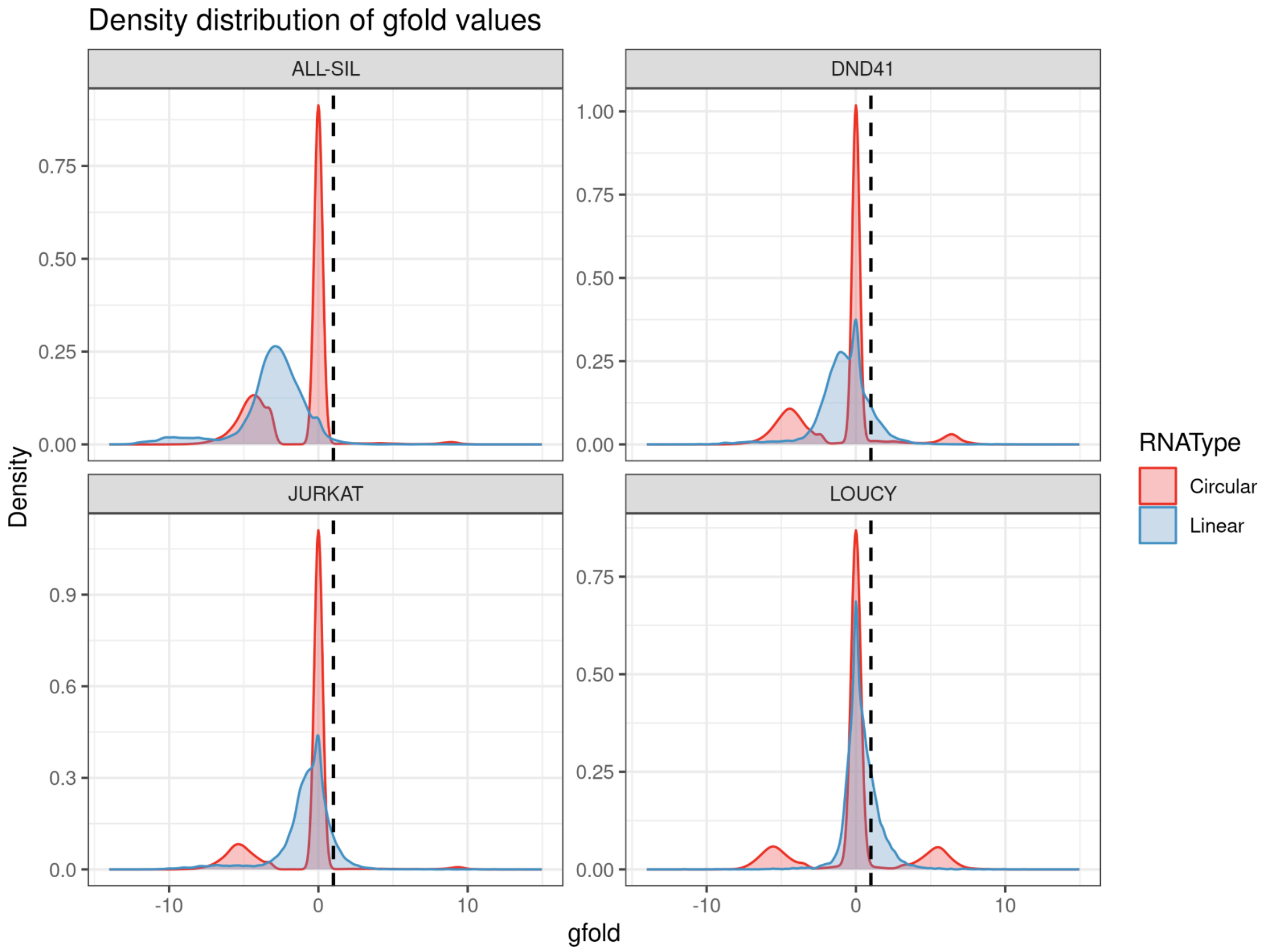

Supplementary Figure S7. Validation of circRNAs.

Sanger sequencing chromatograms showing the backsplice junction of each circRNA, with a schematic representation of their circular structures and included exons. RT-qPCR quantification of circular and linear transcripts in four T-ALL cell lines following RNase R treatment, confirming resistance of circular RNA to exonuclease digestion.

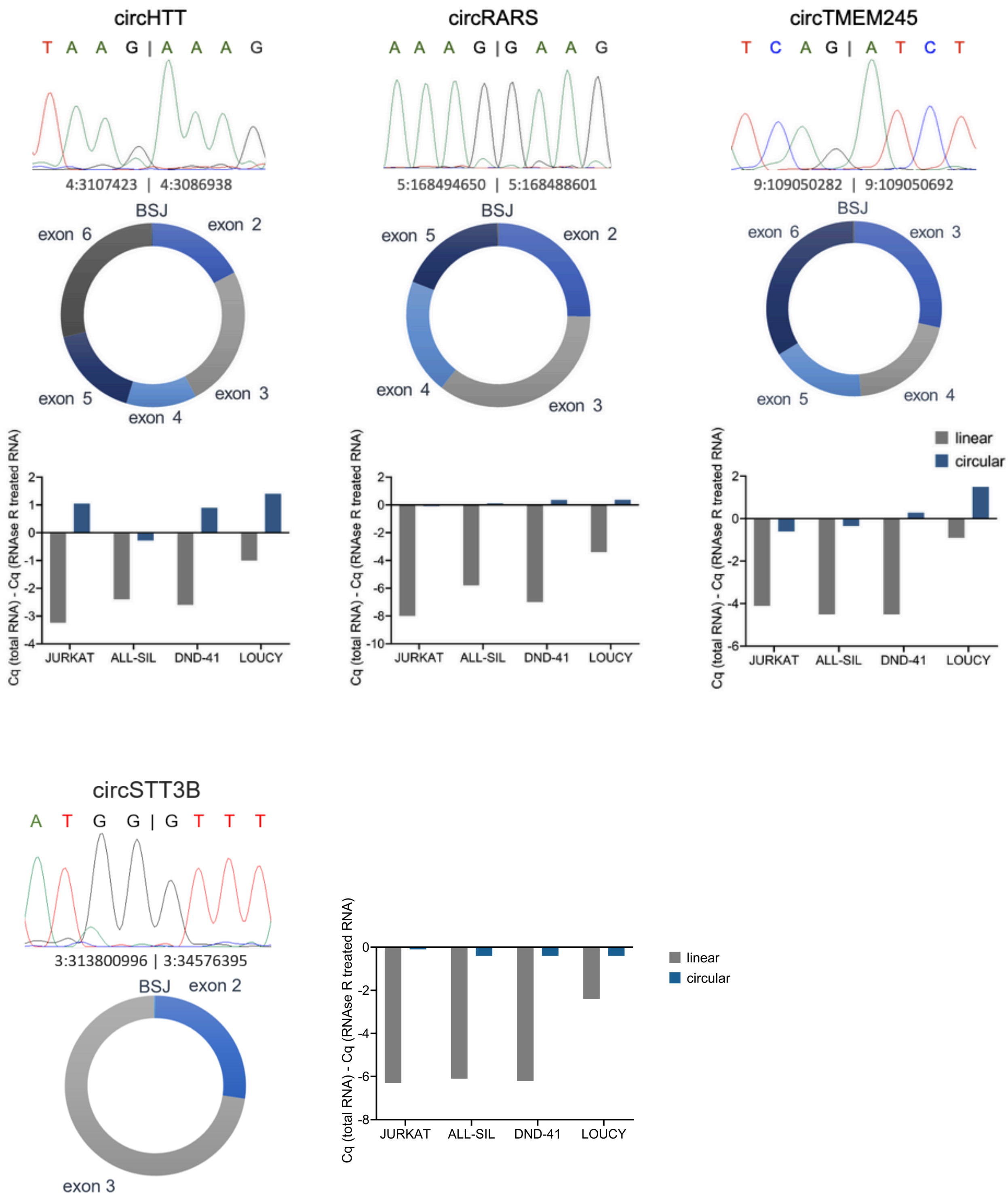

Supplementary Figure S8. Quantification of circN4BP2L2 in the nuclear and cytoplasmic RNA fractions of T-ALL cell lines.

Subcellular fractionation and RT-qPCR analysis of circN4BP2L2 expression in JURKAT (A) and DND-41 (B) cells; *GAPDH* and *ACTB* mRNAs were used as cytoplasmic controls; U3 snoRNA was used as nuclear control. All controls showed the expected localization in each experiment, confirming successful fractionation.

A

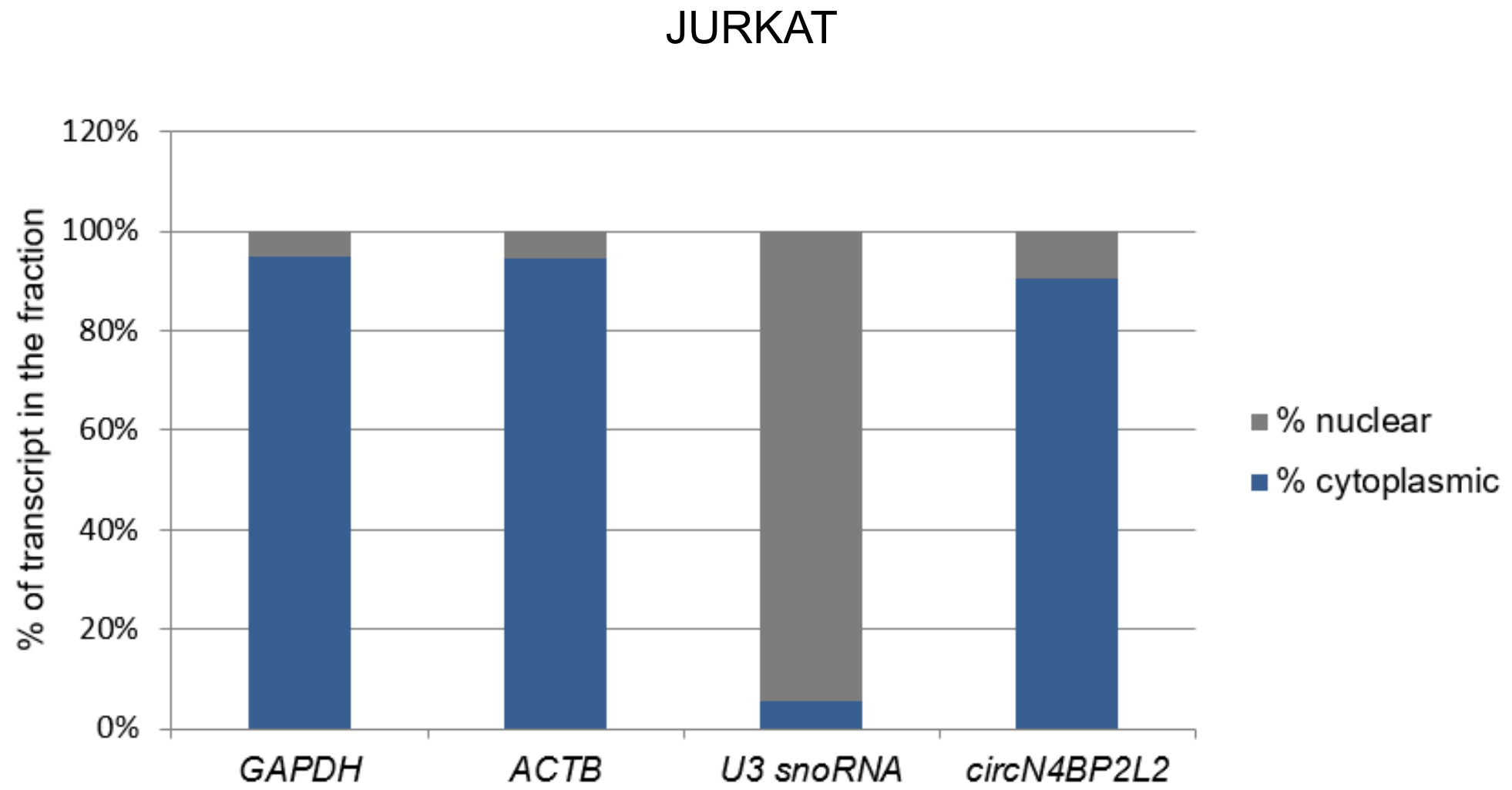

B

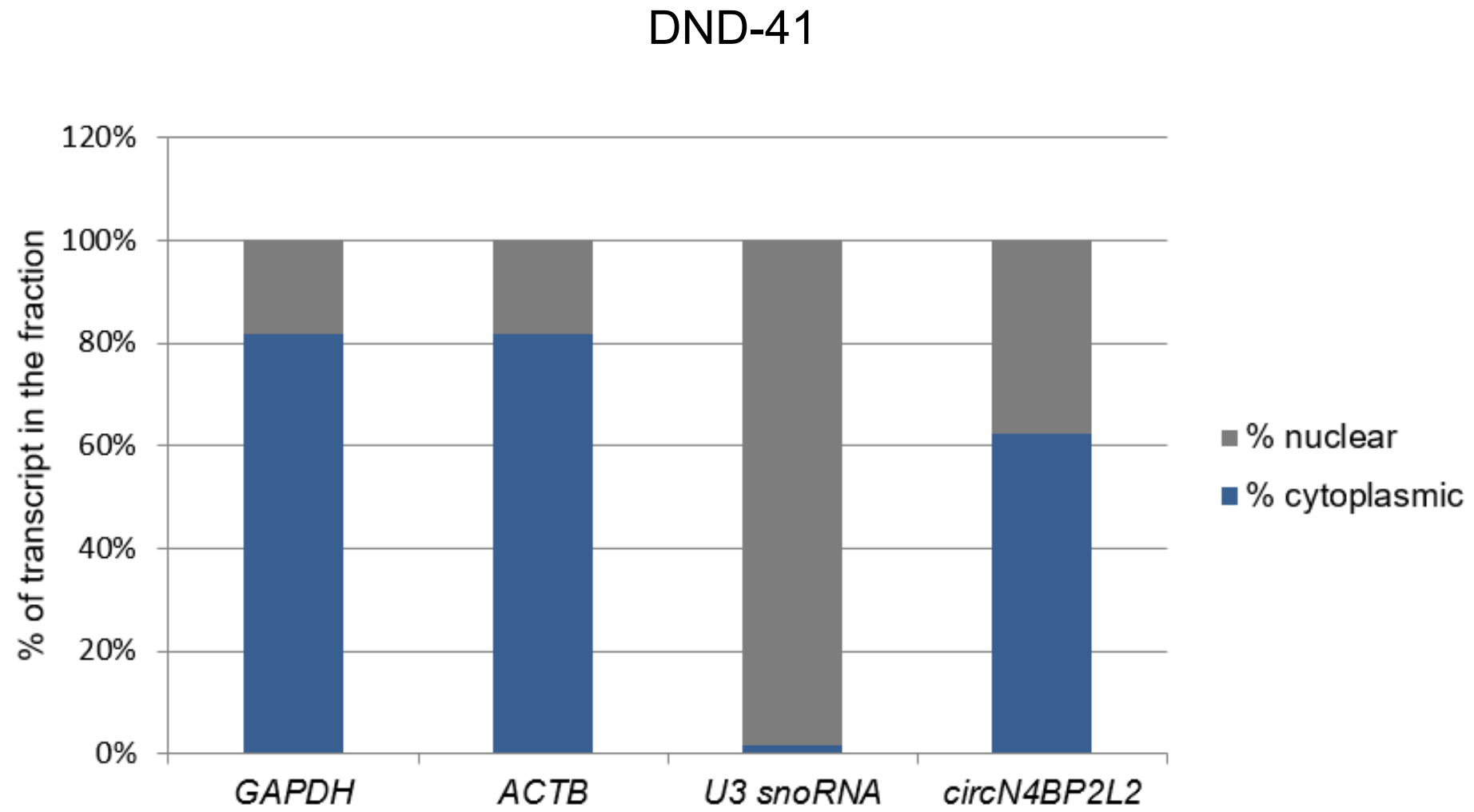

Supplementary Figure S9. Exemplary flow cytometry histograms from Annexin-V APC apoptosis assay upon shRNA-mediated circN4BP2L2 knockdown in JURKAT and DND-41 T-ALL cell lines

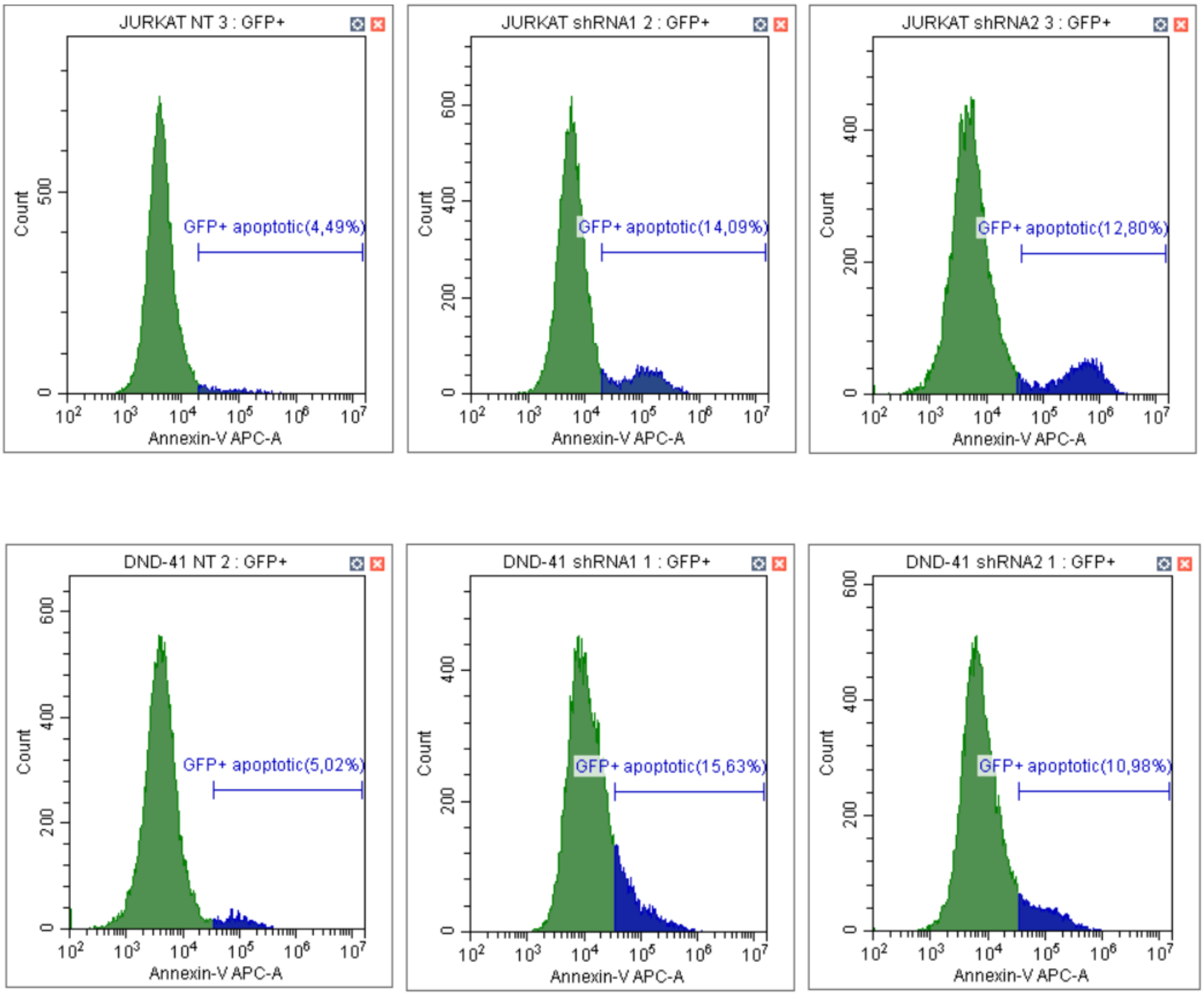

Supplementary Figure S10. Gene Ontology Biological Processes that were significantly enriched upon circN4BP2L2 silencing according to GSEA analysis.

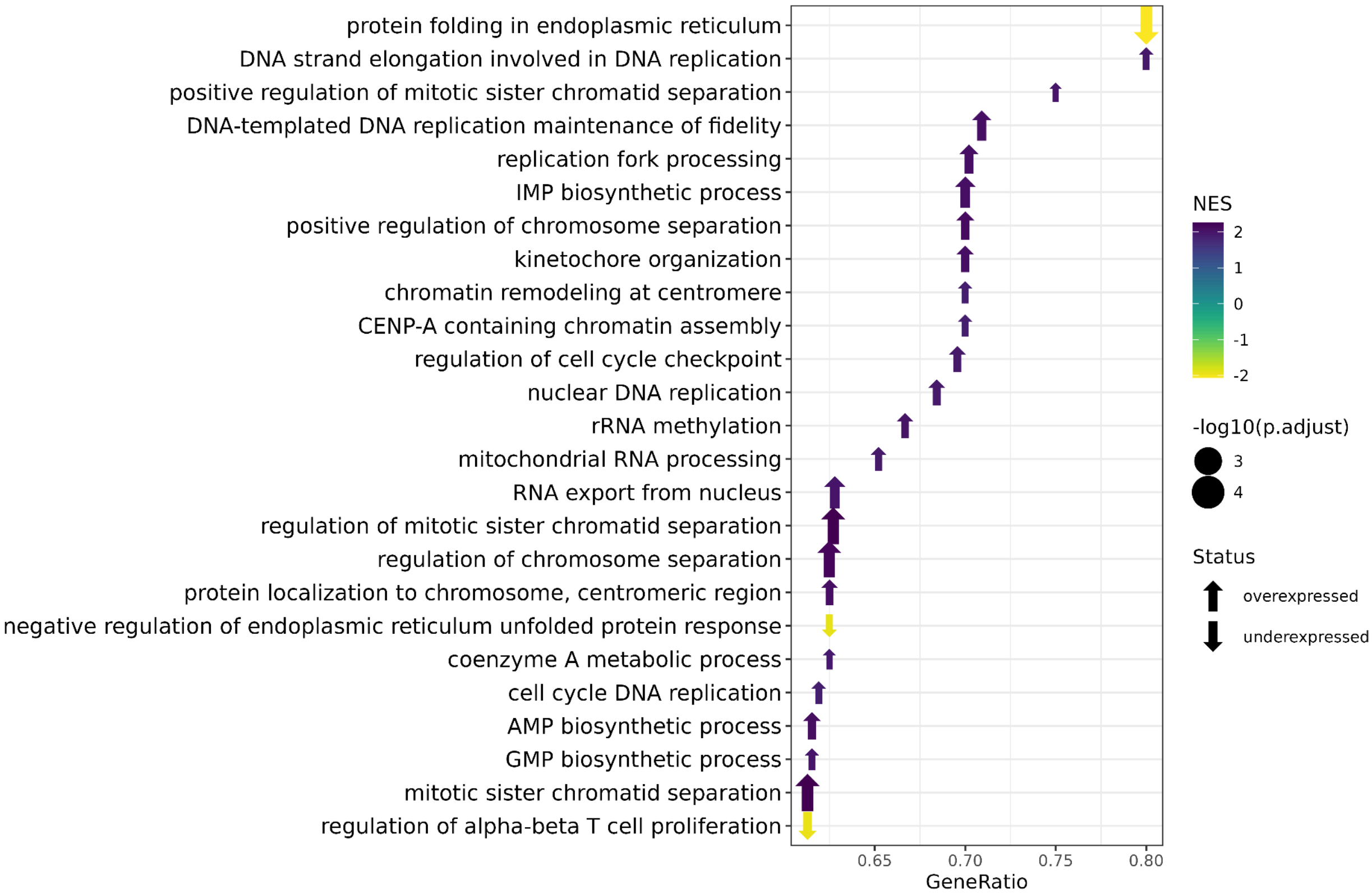

Supplementary Figure S11. CircN4BP2L2 presents predicted binding sites for different miRNAs targeting MYC.

Binding site prediction settings and criteria for selecting validated miRNA-MYC targeting relations are detailed in the Materials and Methods section.

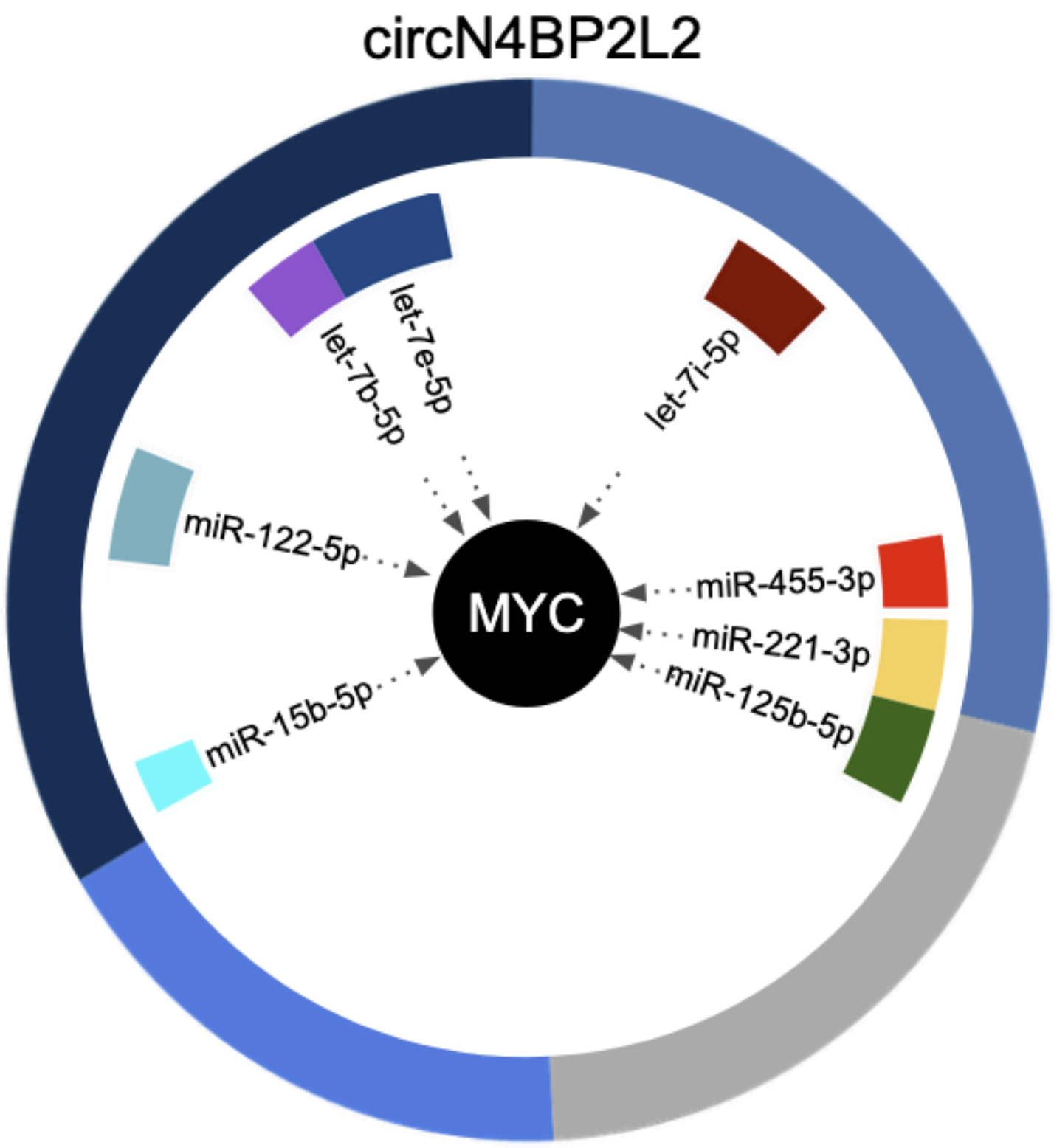

Supplementary Tables

Supplementary Table S1. List of primers used for RT-qPCR detection of studied circRNAs and linear transcripts.

Transcript name	F primer	R primer
circGAPVD1 (9:125337017-125337591:+)	CTGCAGCCATTGGTGCTACT	TGGTGGGCACCTCTCCTCTT
circZNF124 (1:247156405-247159813:-)	GCAATAAGAACTCGGTTGCC	CCTTTGTTTCCTATGGAAGC
circN4BP2L2 (13:32517856-32527532:-)	GCCATAGGAAAAGGATACAG	CATCAGTGCTGAACACAATG
circRARS (5:168488601-168494650:+)	GGGAGAGAATAAAAAGGAAG	CAAACTTGGCCTGCTGACTT
circZNF124 (1:247155565-247159813:-)	CAAGACTTCTAACTCGGTTG	CCTTTGTTTCCTATGGAAGC
circTMEM245 (9:109050282-109050692:-)	AGCCTGACTCCACTATCTCA	ACTCCAAGATCTGATAGCTT
circHTT (4:3086938-3107423:+)	GACAATGAAATTAAGAAAGAAAG	CTGACAGACTGTGCCACTAT
circSTT3B (3:31576395-31580096:+)	CATACTATTTATGGGTTTAAC	AACCCTGGGTAAACAGTACC
GAPVD1	GCAACCTCTGAGGATATTCC	TCAAACATGGCCAGCAGAGA
ZNF124	ACACCCCGGAAGCTGGGAAA	CCTTTGTTTCCTATGGAAGC
N4BP2L2	GTGAAGTCAGAGAAAGATGC	CATCAGTGCTGAACACAATG
RARS	ATGGACGTACTGGTGTCTGA	CAAACTTGGCCTGCTGACTT
TMEM245	CGAGAACATTGAGACATTTC	GATAAAATAGTGTGGTCAGG
HTT	GAGACCCGAAGAATCAGTCCA	CGCTATGAAGGCCTTTAACA
STT3B	GTACCAGGCTACATATCTCG	GTCCAAAAAACTGACCCAGT
ACTB	CTTCCTGGGCATGGAGTCC	ATCTTGATCTTCATTGTGCTG
GAPDH	TGGTCACCAAGGGCTGCTT	AGCTTCCCGTTCTCAGCCTT

Supplementary Table S2. List of primers used for RT-PCR and Sanger sequencing of selected circRNAs.

Transcript name	F primer	R primer
circGAPVD1 (fragment 1)	GAGTAAGGACTCCTCAAGAG	ATGGCCTAGCATAGTGAACC
circGAPVD1 (fragment 2)	TCTGCTGTGGTAAGGCCAAA	TCAAACATGGCCAGCAGAGA
circZNF124 (1:247156405-247159813:-) (fragment 1)	ATCAAACTGGAGATAAACCC	CACATTCCATGCACACATAA
circZNF124 (1:247156405-247159813:-) (fragment 2)	CTGGAGAAAAACGCTATGAA	TTCACAGCCACATCCTCAAA
circZNF124 (1:247155565-247159813:-) (fragment 1)	ATCAAACTGGAGATAAACCC	CACATTCCATGCACACATAA
circZNF124 (1:247155565-247159813:-) (fragment 2)	CTGGAGAAAAACGCTATGAA	TTCACAGCCACATCCTCAAA
circZNF124 (1:247155565-247159813:-) (fragment 3)	CTTGAAAGGACTCACTGAAG	CCTTTGTTTCCTATGGAAGC
circN4BP2L2 (fragment 1)	GCCATAGGAAAAGGATACAG	CATCAGTGCTGAACACAATG
circN4BP2L2 (fragment 2)	GAATTCAGTGGAACCATCAC	TTCCACCAAGTTTCAGGTTC
circRARS (fragment 1)	GTGAATGGAGTTCAACTACC	CAAACTTGGCCTGCTGACTT
circRARS (fragment 2)	TTTGGAAAATCCTCCTCTGC	GTCAATTTCAGCAGTCAGAG
circTMEM245 (fragment 1)	AGCCTGACTCCACTATCTCA	GATAAAATAGTGTGGTCAGG
circTMEM245 (fragment 2)	GGGACAGCCCTTCTCAATTT	ACTCCAAGATCTGATAGCTT
circHTT (fragment 1)	CCTTACCTGGTGAACCTTCT	CTGACAGACTGTGCCACTAT
circHTT (fragment 2)	GAGACCCGAAGAATCAGTCCA	CACGGTCTTTCTTGGTAGCTG
circSTT3B (fragment 1)	GTACCAGGCTACATATCTCG	AACCCTGGGTAAACAGTACC

Supplementary Table S3. List of oligonucleotides used for cloning into shRNA expression vectors.

**Target circRNA**	**Oligonucleotide sequence**	
circGAPVD1<br>(9:125337017-125337591:+)	Sense	GATCCGCAAGAAGAGAGGTGCCCGCCGGCTTCCT GTCAGACTGGTGGGCACCTCTCCTCTTGTTTTTG
	Antisense	AATTCAAAAACAAGAGGAGAGGTGCCCACCAGTCT GACAGGAAGCCGGCGGGCACCTCTCTTCTTGCG
circZNF124<br>(1:247156405-247159813:-)	Sense	GATCCATAAGCAATAAGAACTCGGTTCTCGAGAAC CGAGT TCTTATTGCTTATTTTTTG
	Antisense	AATTCAAAAAATAAGCAATAAGAACTCGGTTCTCGA GAAC CGAGTTCTTATTGCTTATG
circN4BP2L2<br>(13:32517856-32527532:-)	Sense_1	GATCCGGGGAGGCAGAGAATTCTACTCGCTTCCTG TCAGACAAGCAGAATTCTCTGTCTCCCTTTTTG
	Antisense_1	AATTCAAAAAGGGAGACAGAGAATTCTGCTTGTCT GACAGGAAGCGAGTAGAATTCTCTGCCTCCCCG
	Sense_2	GATCCGACAGGAAGACAGAGAATCCTACCTTCCTG TCAGA GCAGAATTCTCTGTCTCCCTGTTTTTTG
	Antisense_2	AATTCAAAAAACAGGGAGACAGAGAATTCTGCTCT GACAG GAAGGTAGGATTCTCTGTCTTCCTGTCG
BamHI sticky end		
EcoRI sticky end		

Supplementary Table S4. List of 144 circRNAs enriched in AGO2-IP in at least three T-ALL cell lines.

CircRNA: Unique identifier for each circRNA; ALL.SIL.AGO2_CPM, DND41.AGO2_CPM, JURKAT.AGO2_CPM, LOUCY.AGO2_CPM: Normalized expression of the circRNA in the AGO2 IP fraction, measured as counts per million (CPM); IALL.SIL.gfold, DND41.gfold, JURKAT.gfold, LOUCY.gfold: gfold of circRNA abundance in AGO2-IP relative to TOT fraction, for each T-ALL cell line.

Provided as an XLS file.

Supplementary Table S5. List of enriched terms upon circN4BP2L2 silencing in JURKAT cells according to GSEA.

NES: normalized enrichment score; p.adjust: FDR-adjusted p-value <= 0.05; GeneRatio: proportion of input genes in the set; SetSize: number of genes in the set; Core enrichment: genes from the dataset contributing to the enrichment.

Provided as an XLS file.

References

1 RNA-seq library preparation for comprehensive transcriptome analysis in cancer cells: The impact of insert size. *Genomics* 2021; **113**: 4149–4162.

2 Gaffo E, Buratin A, Dal Molin A, Bortoluzzi S. Sensitive, reliable and robust circRNA detection from RNA-seq with CirComPara2. *Brief Bioinform* 2022; **23**. doi:10.1093/bib/bbab418.

3 Buratin A, Paganin M, Gaffo E, Dal Molin A, Roels J, Germano G *et al.* Large-scale circular RNA deregulation in T-ALL: unlocking unique ectopic expression of molecular subtypes. *Blood Adv* 2020; **4**: 5902–5914.

4 Cheng J, Metge F, Dieterich C. Specific identification and quantification of circular RNAs from sequencing data. *Bioinformatics* 2016; **32**: 1094–1096.

5 Kim D, Paggi JM, Park C, Bennett C, Salzberg SL. Graph-based genome alignment and genotyping with HISAT2 and HISAT-genotype. *Nat Biotechnol* 2019; **37**: 907–915.

6 Pertea M, Pertea GM, Antonescu CM, Chang T-C, Mendell JT, Salzberg SL. StringTie enables improved reconstruction of a transcriptome from RNA-seq reads. *Nat Biotechnol* 2015; **33**: 290–295.

7 Tarazona S, Furió-Tarí P, Turrà D, Pietro AD, Nueda MJ, Ferrer A *et al.* Data quality aware analysis of differential expression in RNA-seq with NOISeq R/Bioc package.

Nucleic Acids Res 2015; **43**: e140.

8 Feng J, Meyer CA, Wang Q, Liu JS, Shirley Liu X, Zhang Y. GFOLD: a generalized fold change for ranking differentially expressed genes from RNA-seq data. *Bioinformatics* 2012; **28**: 2782–2788.

9 Yu G, Liu Y, Li Z, Deng S, Wu Z, Zhang X *et al.* Genome-wide probing of eukaryotic nascent RNA structure elucidates cotranscriptional folding and its antimutagenic effect. *Nat Commun* 2023; **14**: 5853.

10 Drobna-Śledzińska M, Maćkowska-Maślak N, Jaksik R, Kosmalska M, Szarzyńska B, Lejman M *et al.* Multiomics to investigate the mechanisms contributing to repression of PTPRC and SOCS2 in pediatric T-ALL: Focus on miR-363-3p and promoter methylation. *Genes Chromosomes Cancer* 2022; **61**: 720–733.

11 Love MI, Huber W, Anders S. Moderated estimation of fold change and dispersion for RNA-seq data with DESeq2. *Genome Biol* 2014; **15**: 550.

12 Yu G, Wang L-G, Han Y, He Q-Y. clusterProfiler: an R package for comparing biological themes among gene clusters. *OMICS* 2012; **16**: 284–287.

13 Yu G. *enrichplot*. Bioconductor, 2018 doi:10.18129/B9.BIOC.ENRICHPLOT.

14 Soutschek M, Gross F, Schratt G, Germain P-L. scanMiR: a biochemically based toolkit for versatile and efficient microRNA target prediction. *Bioinformatics* 2022; **38**: 2466–2473.
